# Supplementary material for: Frizzled-Dependent Planar Cell Polarity without Secreted Wnt Ligands
Source: Dev Cell. 2020 Sep 14;54(5):583–592.e5. doi: 10.1016/j.devcel.2020.08.004 (PMC7497783; doi:10.1016/j.devcel.2020.08.004)
Supplement: Document S1. Figures S1–S3 and Table S1 [file mmc1.pdf]

Developmental Cell, Volume *54*

Supplemental Information

Frizzled-Dependent Planar Cell Polarity without Secreted Wnt Ligands

Joyce J.S. Yu, Aude Maugarny-Calès, Stéphane Pelletier, Cyrille Alexandre, Yohanns Bellaiche, Jean-Paul Vincent, and Ian J. McGough

Supplementary Figure 1. Reporters of DWnt expression (Related to main Figure 1D and 2A-B)

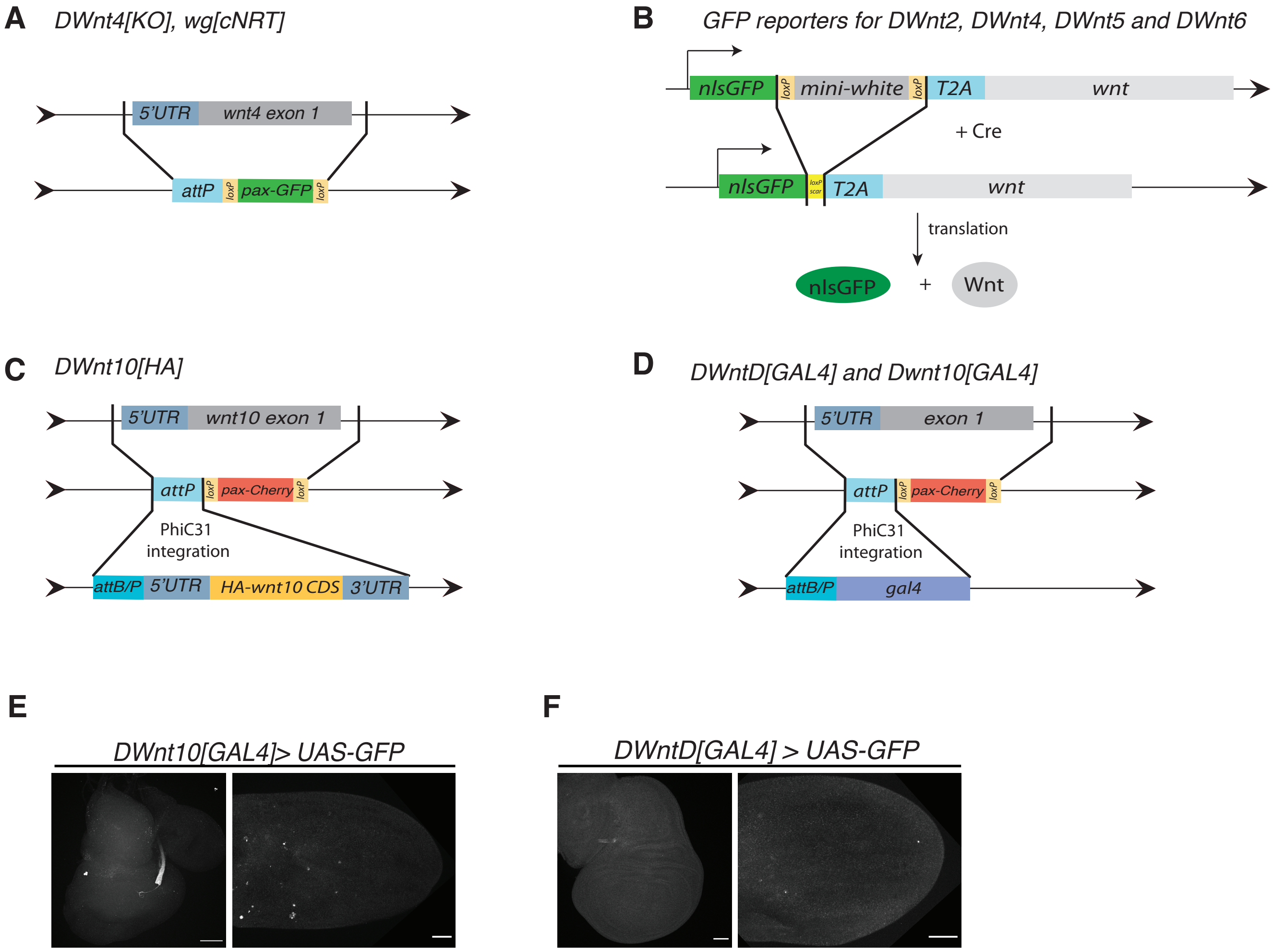

Supplementary Figure 2. A strain for the manipulation of five DWnt genes simunltaneously (Related to main Figure 2C-D)

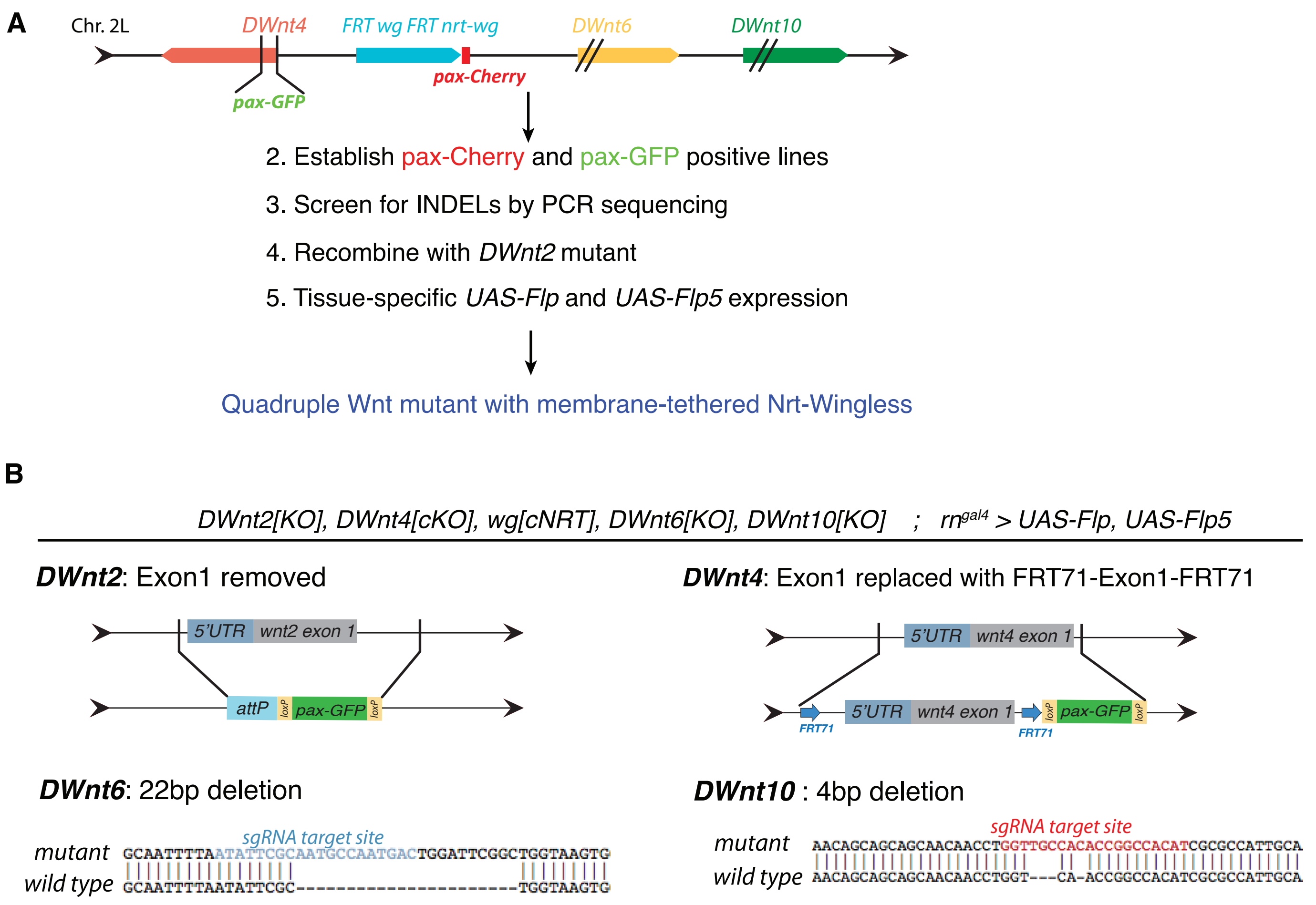

Supplementary Figure 3. Growth and PCP activities of Dsh can be temporally separated (Related to main Figure 3)

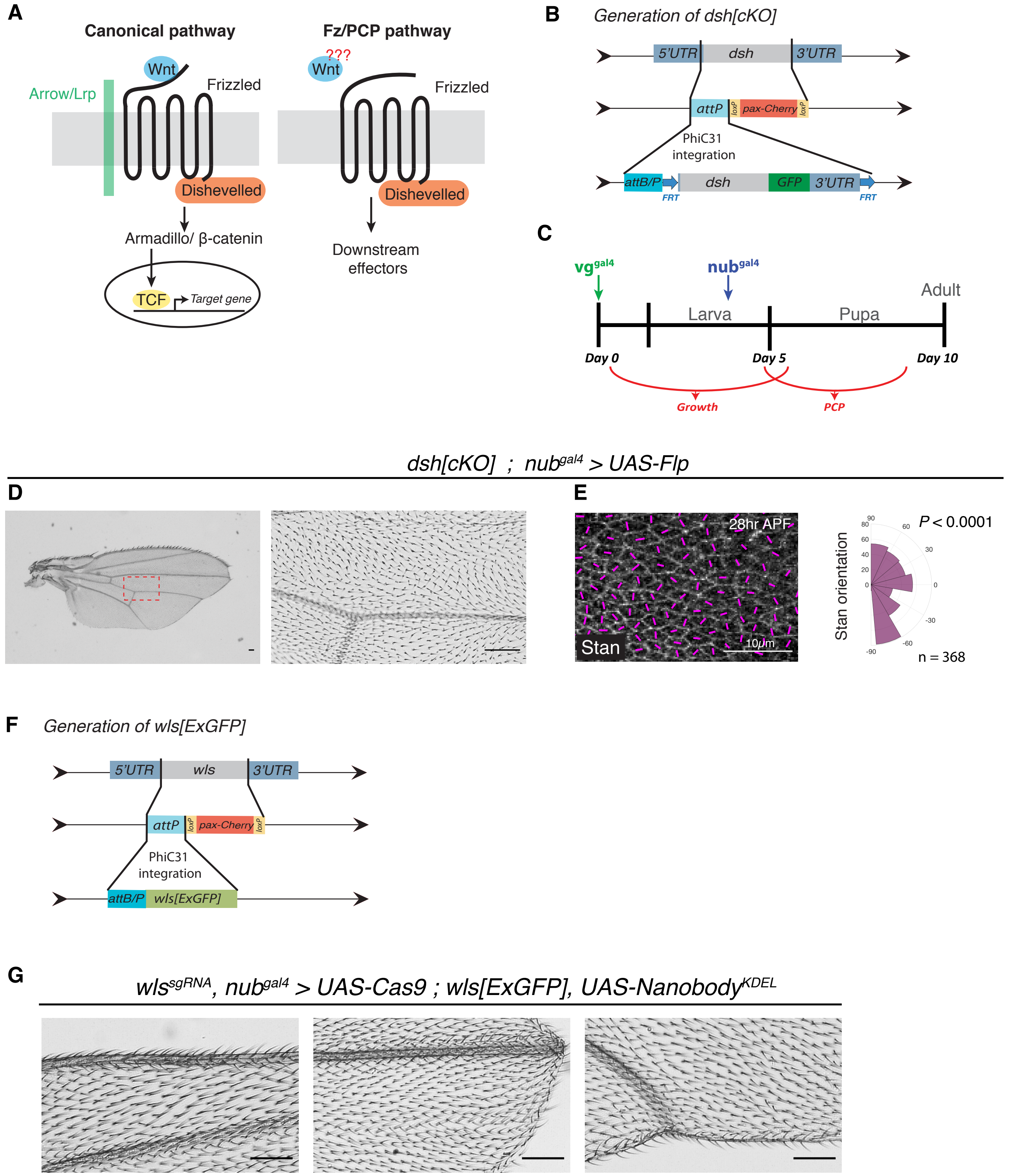

Supp Fig. 1*: Reporters of DWnt expression (related to Figure 1D and 2A-B)*

(**A**) Generation of *DWnt4* mutant by removing the first exon (in the *wg[cNRT]* background). CRISPR target sites (vertical black bars) were chosen in unconserved regions flanking the 5'UTR and first exon. Details for cloning strategies of all transgenic lines generated in this paper are described in the Methods section. (**B**) Generation of nlsGFP-expressing reporters for DWnt2, DWnt4, DWnt5, and DWnt6. (**C**) Generation of *DWnt10[HA]*. (**D**) Generation of Gal4 lines for *DWnt10* and *DWntD*. (**E, F**) Expression patterns of *DWnt10[GAL4]* and *DWntD[GAL4]*, as reporter with *UAS-GFP* expression. Scale bars are 50μm.

Supp Fig 2: *A strain for the manipulation of five DWnt genes simultaneously (related to main Figure 2C-D)*

(**A**) Schematic diagram of the generation of a quadruple DWnt mutants in the *wg[cNRT]* background. The *wg[cNRT]* allele was marked with *pax-Cherry*, which allowed us to exclude multi-genic deletions. The conditional DWnt4 mutant was marked with *pax-GFP*, another selection marker. (**B**) Details of the mutations generated in each *DWnt*. INDELS were created in *DWnt6* and *DWnt10*; the first exon of *DWnt2* was removed; the first exon of DWnt4 was flanked by FRT71 sites, which allowed the tissue specific Flp-excision of the region to create a *DWnt4* mutant.

Supp Fig. 3: *Growth and PCP activities of Dsh can be temporally separated (related to main Figure 3)*

(**A**) Comparison of the canonical Wnt pathway and the Fz/PCP pathway. Fz and Dsh are common components of both pathways. (**B**) Schematic of the generation of the Dsh conditional null allele (*dsh[cKO]*). (**C**) A timeline of Drosophila development showing the approximate periods when growth occurs, and PCP is specified. The time of activation of relevant wing drivers is also shown. (**D, E**) Adult wing and pupal wing Stan polarization upon inactivation of *dsh[cKO] with* nub^{gal4} and *UAS-Flp*. (**F**) Generation of *wls[ExGFP]*, with GFP inserted into the fourth extracellular loop of Wls. (G) Various regions of the same wing showing occasional hair mis-orientation adjacent the edge of the wing.

Supplementary Table 1 (Related to Methods Details)

**Oligonucleotides**	SOURCE	IDENTIFIER
DWnt4[KO] 5'arm forward: CATTAGAATTCAGCGCGTCTAAATGGACAC	This paper	N/A
DWnt4[KO] 5'arm reverse: TAATGGGTACCCTCAAATGCTATTATAATTCTGAAAAGTTTATAATAATC	This paper	N/A
DWnt4[KO] 3'arm forward: CATTAACTAGTGCAATCTATAGGTGACTTTAACAATCAG	This paper	N/A
DWnt4[KO] 3'arm reverse: TAATGACGCGTTGTGTATGTTTTCTCCTCCG	This paper	N/A
DWnt2 nlsGFP reporter 5'arm forward: CCCGGGCTAATTATGGGGTGTCGCCCTTCGCTCAGTTCTTATACGACACTCGCACC	This paper	N/A
DWnt2 nlsGFP reporter 5'arm reverse: GCTCACTACTTTCCGCTTCTTCTTAGGCATCGAGCCCAGCAGCTGGCACTAT	This paper	N/A
DWnt2 nlsGFP reporter 3'arm forward: TGCGGCGACGTGGAGGAGAACCCCGGCCCCATGTGGAAAATACATAACAAGCTCTTAATC	This paper	N/A
DWnt2 nlsGFP reporter 3'arm reverse: GCCCTTGAACTCGATTGACGCTCTTCGGGGAGTTACAAAACTCGTTTAATAATATGTATC	This paper	N/A
DWnt4 nlsGFP reporter 5'arm forward: CCCGGGCTAATTATGGGGTGTCGCCCTTCGTGGGAAAGCGTGAAAACTTCTGGCATC	This paper	N/A
DWnt4 nlsGFP reporter 5'arm reverse: GCTCACTACTTTCCGCTTCTTCTTAGGCATGAAGCGGTTGTTGGTGAATTGTGGATT	This paper	N/A
DWnt4 nlsGFP reporter 3'arm forward: TGCGGCGACGTGGAGGAGAACCCCGGCCCCATGCCATCGCCAACGGGAGTCTTT	This paper	N/A
DWnt4 nlsGFP reporter 3'arm reverse: GCCCTTGAACTCGATTGACGCTCTTCGGGGGATCTGACTTGTTCTAGCCATAAATTGTCG	This paper	N/A
DWnt5 nlsGFP reporter 5'arm forward: CCCGGGCTAATTATGGGGTGTCGCCCTTCGGGTATCTATTATTCGAGGTGGATTAGTG	This paper	N/A
DWnt5 nlsGFP reporter 5'arm reverse: AACAATACTCTGTAATAGTAATAGTAAGAGATGCCTAAGAAGAAGCGGAAAGTAGTGAGC	This paper	N/A
DWnt5 nlsGFP reporter 3'arm forward: TGCGGCGACGTGGAGGAGAACCCCGGCCCCATGAGTTGCTACAGAAAAAGGCACTTTCT	This paper	N/A
DWnt5 nlsGFP reporter 3'arm reverse: GCCCTTGAACTCGATTGACGCTCTTCGGGGGTGTCTCGTTGAGCAGTATCACCTG	This paper	N/A
DWnt6 nlsGFP reporter 5'arm forward: CCCGGGCTAATTATGGGGTGTCGCCCTTCGCAGATCCATATGAACGGTCAGCGC	This paper	N/A
DWnt6 nlsGFP reporter 5'arm reverse:	This paper	N/A

GCTCACTACTTTCCGCTTCTTCTTAGGCATAGCCGCCAAATGCAGCGGCAACAA		
DWnt6 nlsGFP reporter 3’arm forward: TGCGGCGACGTGGAGGAGAACCCCGGCCCCATGCGTTTGCTCATGGTAATTGCAATTTTA	This paper	N/A
DWnt6 nlsGFP reporter 3’arm reverse: GCCCTTGAACTCGATTGACGCTCTTCGGGGCCTCCGCGGTTTGTTTATTTATTTATTCAT	This paper	N/A
DWnt10[KO]^{attP} 5’arm forward: CATTAGAATTCAGCTCGGCTCTCGTCTCATAG	This paper	N/A
DWnt10[KO]^{attP} 5’arm reverse: TAATGGGTACCAAAGCATGGAAATTAAGGGAAATATAC	This paper	N/A
DWnt10[KO]^{attP} 3’arm forward: CATTAGACGTCAGAAGATGTTCAGAGGCAGAC	This paper	N/A
DWnt10[KO]^{attP} 3’arm reverse: TAATGACCGGTTAACTAAAACTAAGGCGATGTACTCATG	This paper	N/A
DWnt10[HA] forward: CATTAGAATTCTCATAGTCGCGCGACTCG	This paper	N/A
DWnt10[HA] reverse: TAATGGGTACCGACAGACTAATTATGCATCTTATTGTTCGTATTTACAAAATATATTT	This paper	N/A
DWntD[KO]^{attP} 5’arm forward: CATTAGCGGCCGCATGTAGTGGGGGAATCTCAAG	This paper	N/A
DWntD[KO]^{attP} 5’arm reverse: TAATGGGTACCACACTTATATAGCCTGCAAATCCC	This paper	N/A
DWntD[KO]^{attP} 3’arm forward: CATTAGACGTCCTACAGGTGGTTTAATAATTACGGATTTTAGAGT	This paper	N/A
DWntD[KO]^{attP} 3’arm reverse: TAATGACGCGTCTGATGCAGCGGCCAC	This paper	N/A
DWnt4[cKO] 5’arm forward: GCGAATTGGAGCTGAATTCCTGTAGATAAACCCTTGGGG	This paper	N/A
DWnt4[cKO] 5’arm reverse: TCTAGAAAGTATAGAAACTTCAGGTGACTTTAACAATCAGGTGATTG	This paper	N/A
DWnt4[cKO] exon1 forward: GAAGTTTCTATACTTTCTAGAGAATAGAAACTTCATCGATTGCATTTTGCTCCTAATATCTTCAATTTCCACACATTTTTCATTTGAGA	This paper	N/A
DWnt4[cKO] exon1 reverse: GTTGGGGCACTACGGGTACCGAAGTTTCTATTCTCTAGAAAGTATAGAAACTTCAGCATTTGAGGACGGCTAACATGAAATCAATATTTTCGATTTCTAGAATTTTGCC	This paper	N/A
DWnt4[cKO] 3’arm forward: CATTAACTAGTATTATAATTCTGAAAAGTTTATAATAATCGCTTCCTG	This paper	N/A
DWnt4[cKO] 3’arm reverse: TAATGACGCGTGCTTCAAGCTTATACGCACAGAA	This paper	N/A
DWnt2[KO] 5’arm forward: CATTAGCGGCCGCATTATCTGGAATTTAAAAGTCTTGGCACAC	This paper	N/A
DWnt2[KO] 5’arm reverse: TAATGGGTACCGTACTACTACTATCCAACTTTCCACTCT	This paper	N/A

DWnt2[KO] 3’arm forward: CATTAACGCGTGTATTTTGATTTTGGTGAAATGCTTGAAAAG	This paper	N/A
DWnt2[KO] 3’arm reverse: TAATGACCGGTCATAAAGGCTGTTCAACATGCC	This paper	N/A
dsh[KO]^{attP} 5’arm forward: ATGCAGGCGGCCGCTATCAGCCGTCGTGCGTG	This paper	N/A
dsh[KO]^{attP} 5’arm reverse: TGACTCACATATGCCTCGACGCGATAAAACGCGATC	This paper	N/A
dsh[KO]^{attP} 3’arm forward: TGCACCTAGGTGAGATTGGTTTCTTTAACGCATTTTAGCTG	This paper	N/A
dsh[KO]^{attP} 3’arm reverse: CGTCAACCGGTCATTTCGTATTAGTTGTATGTACGGAAGTTGAC	This paper	N/A
RIV10$^{dsh\text{-}GFP}$ forward: ATGGTCAGCTAGCGATCGCGTTTTATCGCGTCGAGGAGTTTTCCCGTGGATTTCCGCAGTCGGCGCAGCTAAA	This paper	N/A
RIV10$^{dsh\text{-}GFP}$ reverse: TCCATGACCGGTCCATGCCCGGAATGTTTCCG	This paper	N/A
pBS-KS$^{dsh\text{-}GFP}$ forward: CGTATCCGTATTTAATTACGTATTGGGAGGTTCGGGAGGTGGTTCGGGAGGTGGAGGTTCGGGAGTGAGCAAGGGCGAGGA	This paper	N/A
pBS-KS$^{dsh\text{-}GFP}$ reverse: GAAAGGTTTCTACAATACgtaCTTGTACAGCTCGTCCATGCC	This paper	N/A
wls[ExGFP] forward: TGACGACGTCGTGAGCAAGGGCGAGGAGCTGTTCA	This paper	N/A
wls[ExGFP] reverse: GTCGACGTCCTTGTACAGCTCGTCCATGCCGAG	This paper	N/A
