# Supplementary material for: Frizzled-Dependent Planar Cell Polarity without Secreted Wnt Ligands
Source: Dev Cell. 2020 Sep 14;54(5):583–592.e5. doi: 10.1016/j.devcel.2020.08.004 (PMC7497783; doi:10.1016/j.devcel.2020.08.004)
Supplement: Document S2. Article plus Supplemental Information [file mmc2.pdf]

Short Article

Developmental Cell

Frizzled-Dependent Planar Cell Polarity without Secreted Wnt Ligands

Graphical Abstract

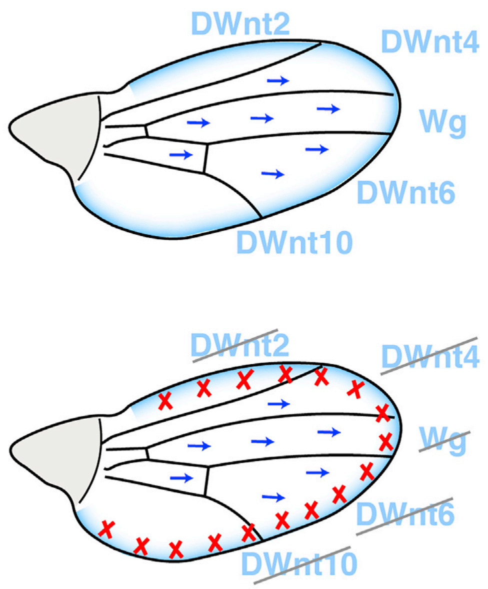

Highlights

- Reporter knockin alleles identify all the Wnt genes expressed in the *Drosophila* wing
- A quintuple mutant removes all diffusible DWnts from the wing but leaves PCP unperturbed
- The secretion and release of Wnts is dispensable for *Drosophila* wing PCP establishment
- The wing margin does not provide a global cue for PCP

Authors

Joyce J.S. Yu, Aude Maugarny-Calès, Stéphane Pelletier, Cyrille Alexandre, Yohanns Bellaiche, Jean-Paul Vincent, Ian J. McGough

Correspondence

jp.vincent@crick.ac.uk (J.-P.V.), ian.mcgough@crick.ac.uk (I.J.M.)

In Brief

Planar cell polarity (PCP) requires Frizzled, which transduces Wnt signaling. Yu et al show that the secretion and release of Wnts is dispensable for proper PCP establishment in the *Drosophila* wing. The global cue organizing PCP in this tissue remains unknown but does not originate from the wing margin.

Yu et al., 2020, Developmental Cell *54*, 583–592
September 14, 2020 © 2020 The Author(s). Published by Elsevier Inc.
https://doi.org/10.1016/j.devcel.2020.08.004

Developmental Cell

CellPress
OPEN ACCESS

Short Article

Frizzled-Dependent Planar Cell Polarity without Secreted Wnt Ligands

Joyce J.S. Yu,[1] Aude Maugarny-Calès,[2,3] Stéphane Pelletier,[2,3] Cyrille Alexandre,[1] Yohanns Bellaiche,[2,3] Jean-Paul Vincent,[1,4,*] and Ian J. McGough[1,*]
[1]The Francis Crick Institute, London NW1 1AT, UK
[2]Institut Curie, PSL Research University, CNRS UMR 3215, INSERM U934, 75248 Paris Cedex 05, France
[3]Sorbonne University, CNRS UMR 3215, INSERM U934, 75005 Paris, France
[4]Lead Contact
*Correspondence: jp.vincent@crick.ac.uk (J.-P.V.), ian.mcgough@crick.ac.uk (I.J.M.)
https://doi.org/10.1016/j.devcel.2020.08.004

SUMMARY

Planar cell polarity (PCP) organizes the orientation of cellular protrusions and migratory activity within the tissue plane. PCP establishment involves the subcellular polarization of core PCP components. It has been suggested that Wnt gradients could provide a global cue that coordinates local PCP with tissue axes. Here, we dissect the role of Wnt ligands in the orientation of hairs of *Drosophila* wings, an established system for the study of PCP. We found that PCP was normal in quintuple mutant wings that rely solely on the membrane-tethered Wingless for Wnt signaling, suggesting that a Wnt gradient is not required. We then used a nanobody-based approach to trap Wntless in the endoplasmic reticulum, and hence prevent all Wnt secretion, specifically during the period of PCP establishment. PCP was still established. We conclude that, even though Wnt ligands could contribute to PCP, they are not essential, and another global cue must exist for tissue-wide polarization.

INTRODUCTION

Planar cell polarity (PCP) refers to the polarity that epithelial cells acquire along the plane of the epithelium, orthogonal to the apical-basal axis. In a wide range of metazoans, PCP contributes to cell proliferation, cell fate decisions, body axis elongation, and morphogenesis, as well as to the orientation of cellular protrusions, such as hairs (Goodrich and Strutt, 2011; Adler, 2012; Yang and Mlodzik, 2015; Butler and Wallingford, 2017; Lawrence and Casal, 2013). Moreover, aberrant PCP has been linked to diseases, such as polycystic kidney disease, deafness, and cancer (Yates et al., 2010; Lu and Sipe, 2016; VanderVorst et al., 2018). Understanding the molecular basis of PCP establishment and maintenance is, therefore, important from both a fundamental and a biomedical perspective. Genetic analyses have identified many conserved molecules that mediate PCP from flies to humans. A number of these proteins make up the so-called core PCP pathway. They include, for example, the transmembrane proteins Frizzled (Fz1, FZD in vertebrates) and Starry Night (Stan aka Flamingo, CELSR in vertebrates) and the cytoplasmic protein Dishevelled (Dsh, DVL in vertebrates). A second PCP pathway, named after two of its components Fat (Ft) and Dachsous (Ds), has been identified in *Drosophila* (Mahoney et al., 1991; Clark et al., 1995; Zeidler et al., 1999). Its involvement in vertebrates is less well characterized than in flies, and its relationship with the core pathway remains controversial (Thomas and Strutt, 2012; Matis and Axelrod, 2013). However, the finding that, in some tissues, this pathway can drive PCP in the absence of the core pathway (Casal et al., 2006) shows that cells can use multiple inputs to orient themselves.

Core PCP relies on the complementary localization of components at the distal and proximal sides of cells. These components include transmembrane proteins, which mediate cell interactions that coordinate polarity locally, and intracellular factors, which stabilize the asymmetry within each cell (Vinson et al., 1989; Klingensmith et al., 1994; Taylor et al., 1998; Wolff and Rubin, 1998; Usui et al., 1999; Chae et al., 1999; Wallingford et al., 2000; Feiguin et al., 2001; Strutt, 2001; Axelrod, 2001; Tree et al., 2002; Jenny et al., 2003; Bastock et al., 2003; Jenny et al., 2005; Devenport and Fuchs, 2008; Strutt et al., 2011). In addition, it is thought that tissue-wide global cues provide an overall direction to PCP relative to embryonic axes. One attractive possibility is that this is achieved by morphogen gradients, although this has not been directly demonstrated. Among the key components of core PCP are Frizzled proteins, which are transmembrane proteins that bind Wnts through their extracellular cysteine rich domain (CRD) (Bhanot et al., 1996; Hsieh et al., 1999). Indeed, Frizzled receptors are key mediators of canonical Wnt signaling. Thus, it is conceivable that Frizzled proteins could read and transduce a Wnt gradient into PCP. It has been suggested, therefore, that a long-range Wnt gradient would bias Frizzled activity and kickstart the molecular interactions that stabilize the asymmetric distribution of other core PCP

This is an open access article under the CC BY license (http://creativecommons.org/licenses/by/4.0/).

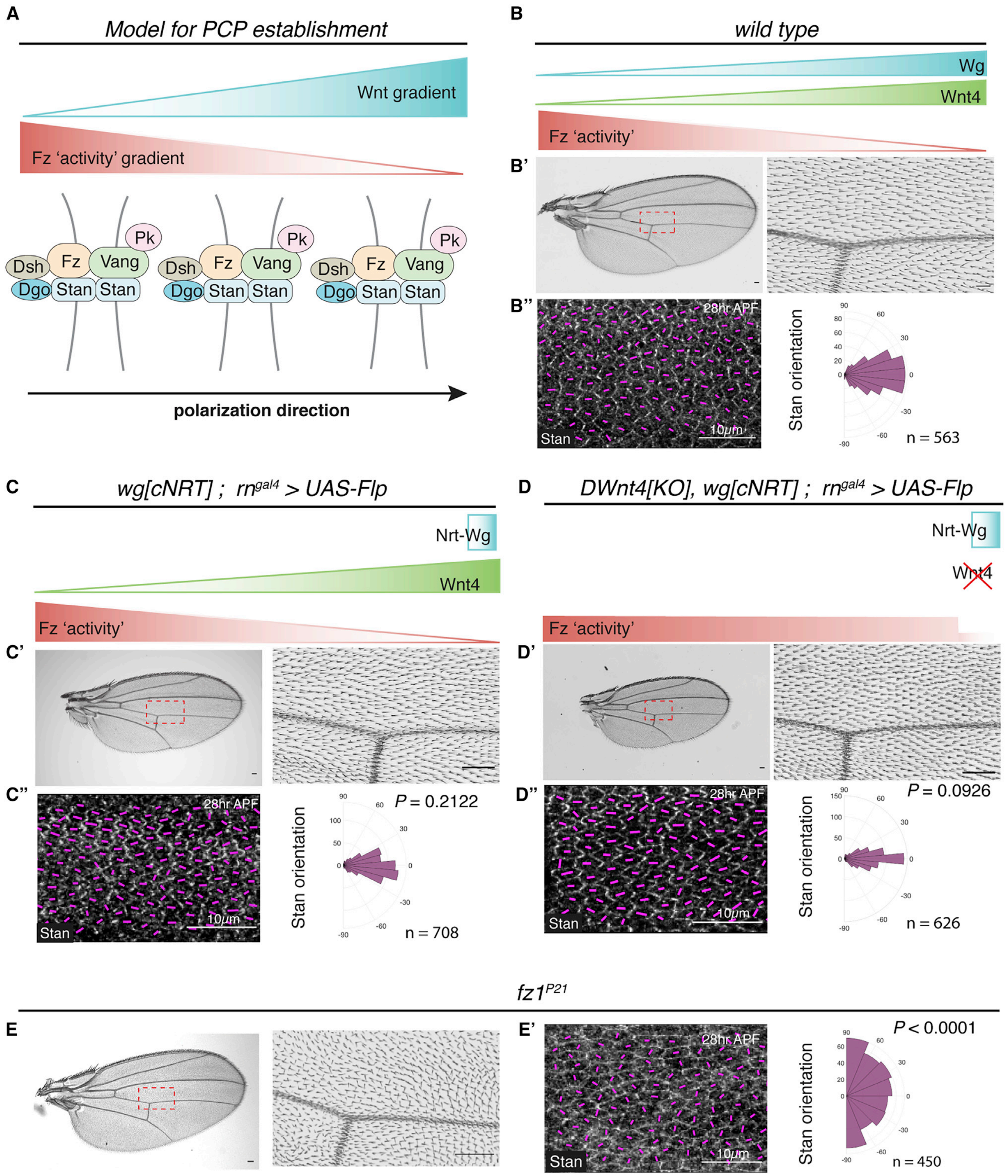

Figure 1. PCP Is Normal with Membrane-Tethered Wingless and the Absence of DWnt4

(A) A model of how a Wnt gradient may lead to a Fz "activity" gradient that directs the asymmetric localization of core PCP protein complexes, eventually polarizing hair outgrowth in one direction. Note that PCP is initially radial toward the prospective wing margin, where Wg and Wnt4 are expressed. The PCP axis subsequently reorient to the final proximal-distal pattern during morphogenesis (Aigouy et al., 2010). For simplicity, the Wnt gradient and PCP are shown aligned with the proximal-distal axis throughout.

(legend continued on next page)

components and the morphological implementation of PCP (Figure 1A) (Gubb and García-Bellido, 1982; Adler et al., 1997; Strutt, 2001; Struhl et al., 2012; Fisher and Strutt, 2019). Although attractive, this scenario remains a model since an endogenous Fz activity gradient has not been formally demonstrated.

A number of studies have implicated Wnt ligands in PCP establishment. In fish and frog embryos, mutation or knockdown of Wnt5a or Wnt11 lead to a reduction in the PCP-dependent cell movements that lead to axis elongation (Rauch et al., 1997; Heisenberg et al., 2000; Tada and Smith, 2000; Wallingford et al., 2001; Andre et al., 2015). Likewise, in the mouse limb bud, the deletion of Wnt5a interferes with the establishment of PCP in chondrocytes along the proximal-distal axis, and thus with tissue elongation (Gao et al., 2011; Yang et al., 2017). Therefore, in these instances, a Wnt ligand is required for PCP, although it is not clear whether this role is instructive or permissive (Gao, 2012; Lawrence and Casal, 2013). In fish and frog embryos, the PCP phenotypes caused by Wnt inhibition can be rescued by the uniform expression of exogenous Wnts, suggesting that a graded ligand distribution may not be required for PCP. Nevertheless, localized ectopic Wnt expression can orient PCP both in *Xenopus* mesoderm and the mouse limb bud (Chu and Sokol, 2016; Minegishi et al., 2017). Therefore, in these systems, a Wnt gradient can provide information for polarization, even though it may not be required.

Because of a wealth of genetic tools and a long history of PCP research, *Drosophila* is well suited to investigate the requirement of Wnt gradients in PCP. In *Drosophila*, PCP is readily assayed by measuring the orientation of hairs that decorate much of the cuticle. For example, at the surface of developing wings, the core PCP pathway orients actin protrusions, which serve as a template for the formation of hairs during metamorphosis (Wong and Adler, 1993). In the wild type, these protrusions point distally but can be reoriented by the ectopic expression of Wingless or DWnt4 (Wu et al., 2013). Therefore, as in vertebrates, the gain-of-function evidence suggests that a Wnt gradient is sufficient to reorient hairs. Loss-of-function tests are difficult to perform and interpret because of the requirement of Wingless for wing specification and growth, which occur before PCP establishment (Ng et al., 1996). Nevertheless, it has been possible to interfere with Wingless and DWnt4 with a combination of hypomorphic alleles compatible with growth. Adult flies were rarely recovered, but in most pupal wings, the pre-hair actin bundles were found to be partially misoriented (Wu et al., 2013). This observation suggests that, in the developing wing, Wingless and DWnt4 are needed redundantly for the establishment of PCP, although it does not directly address whether these Wnts must be graded. However, in another tissue, the adult abdomen, the possibility that Wnts could provide an instructive cue that orients bristles has been ruled out (Lawrence et al., 2002; Casal et al., 2006). Therefore, the requirement of Wnts, and/or the instructive value of their graded distribution for PCP remains a matter of debate both in flies and vertebrates.

The *Drosophila* genome encodes seven Wnts. Six of them carry a palmitoleate moiety that is essential for engagement with the Frizzled CRD and have a well-defined vertebrate ortholog (in parenthesis below): Wingless (Wnt1), DWnt2 (Wnt7), DWnt4 (Wnt9), DWnt 5 (Wnt5), DWnt6 (Wnt6), and DWnt10 (Wnt10) (Nusse, 2020). *Drosophila* also encodes a non-conserved WntD, which is not palmitoleoylated, and therefore, is unlikely to bind the CRD of Fz (Wu and Nusse, 2002). We took advantage of the recent developments in genome engineering to create a panel of alleles, some conditional, in all the genes encoding *Drosophila* Wnts. With these genetic tools, we found that PCP can be established in the absence of a diffusion-based Wnt gradient. In further analysis, we show that the Frizzled-dependent core PCP pathway does not need any Wnt ligand to organize PCP in the developing wing. Therefore, in this instance, another non-Wnt global cue must control the subcellular asymmetry of Fz and other core PCP components.

RESULTS

DWnt4 Is Not Required for PCP, Even in the Absence of Diffusible Wingless

Larvae expressing membrane-tethered (non-diffusible) Wingless (NRT-Wg) from the endogenous locus can develop into flies with apparently normal appendages, albeit with a delay and at a reduced frequency (Alexandre et al., 2014). Wing hairs in these animals appear to be normally oriented, suggesting that PCP establishment does not require a Wingless gradient. To improve viability and sample recovery, we took advantage of an allele, here, referred to as *wg*[*cNRT*], which can be converted in a tissue-specific manner from expressing wild-type Wg to expressing NRT-Wg. Allele conversion was induced with *UAS-Flp* and rn^{gal4}, which is expressed specifically in wing primordia at the second instar stage (St Pierre et al., 2002). The resulting pupal wings, which presumably lack diffusible Wingless, were stained for Stan (aka Fmi), an atypical cadherin that forms homophilic bridges across the proximal-distal cell junctions, and thus, serves as an early marker of PCP (Lu et al., 1999; Usui et al., 1999). The distribution of Stan was indistinguishable from that in wild-type pupae (Figures 1B and 1C), confirming that diffusible Wingless is not necessary for PCP. It has been suggested that a DWnt4 gradient could redundantly promote PCP in the *Drosophila* wing (Wu et al., 2013). To probe this suggestion, we generated a *DWnt4* mutant (*DWnt4[KO]*) in the *wg*[*cNRT*] background (Figure S1A). The resulting flies were used to create

(B) A wild-type adult wing (B′) and a 28 h APF pupal wing with anti-Stan staining (B″). Here and in subsequent figures, the orientation and magnitude of Stan's asymmetric localization in each cell is depicted by magenta lines. Stan orientation data from the pupal wing region (corresponding to the region marked by a red rectangle in the adult wing) was tabulated on a polar coordinate histogram, with n denoting the number of cells (pooled from 4 pupal wings).

(C and D) Wing, pupal wing, and Stan orientation polar histogram for homozygous NRT-Wg larvae (no diffusible Wingless) (C) or for homozygous NRT-Wg larvae lacking DWnt4 (D). Conversion to NRT-Wg was triggered by GAL4-driven Flp, which has been shown to be efficient previously (Hadjieconomou et al., 2011; Bosch et al., 2017). The p values were calculated with a two-sample Kolmogorov-Smirnov test that compares the distribution of the Stan orientation in mutant conditions (C″ and D″) with that in the wild type (shown in B″).

(E) Wing from a hemizygous $fz1^{P21}$ mutant fly.

(E') Pupal wing and polar histogram of Stan orientations for the same genotype. Scale bars are 50 μm unless specified otherwise.

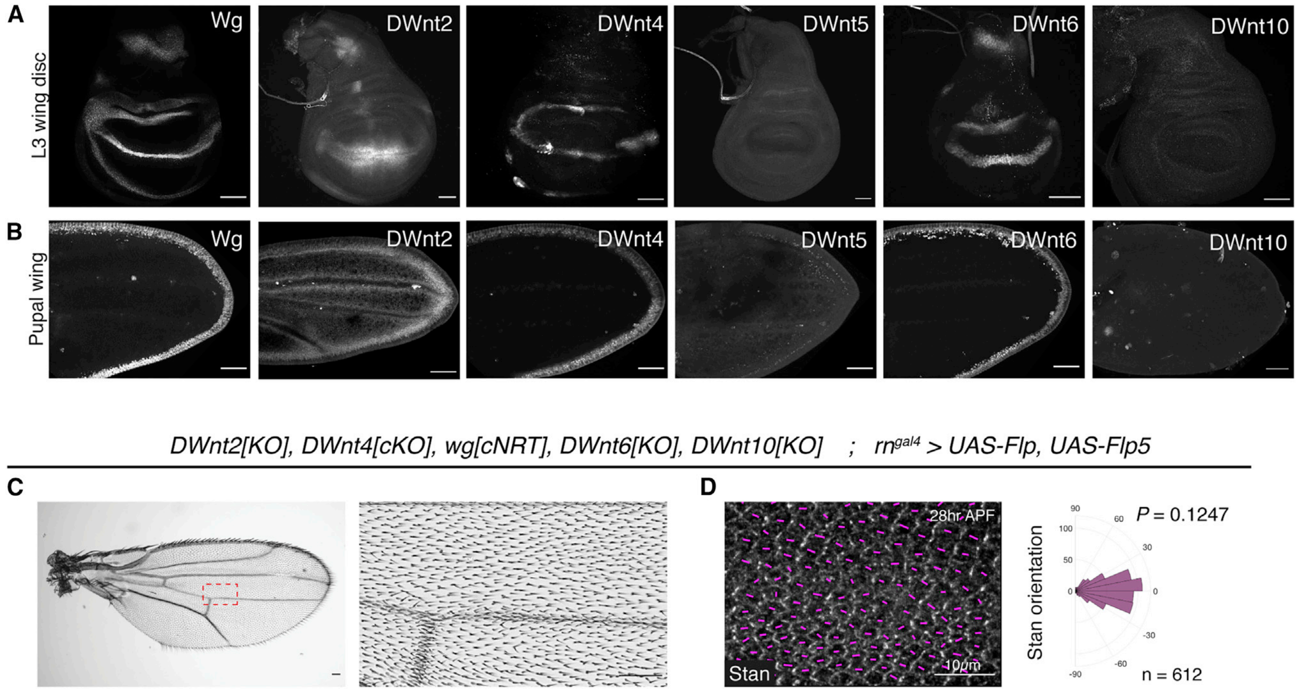

Figure 2. Deletion of DWnt2, DWnt4, DWnt6 and DWnt10 in a Membrane-Tethered Wingless Background Does Not Impair PCP

(A and B) Expression patterns of DWnts in third-instar wing discs and pupal wings reveal all the expressed DWnts.

(C) Wing derived from primordia expressing NRT-Wg instead of Wg, and lacking DWnt2, DWnt4, DWnt6, and DWnt10.

(D) Pupal wings and polar histogram of Stan orientations for the same genotype. The p value was calculated using the two-sample Kolmogorov-Smirnov test to compare the distribution of Stan orientation of the mutant condition (D) to that of the wild type in Figure 1B″. Scale bars are 50 μm unless specified otherwise.

NRT-Wg-expressing wing primordia lacking all DWnt4 proteins. PCP, as determined by Stan localization, was still normal (Figure 1D). For comparison, significant polarity defects were seen in *fz1^P21^* mutants, as expected, since Fz1 is the only Frizzled receptor required for PCP in *Drosophila* (Figures 1E and E'). We conclude that gradients of diffusible Wingless and DWnt4 are dispensable for PCP, but we cannot exclude a possible neomorphic activity of NRT-Wg on Fz1 activity or a contribution of other DWnts.

Multiple Wnts Are Expressed in the Developing Wing

To identify the Wnts that are expressed in wing primordia and hence possibly involved in PCP in this tissue, we generated a panel of reporter genes. CRISPR-Cas9 was used to insert DNA fragments encoding nuclear-targeted GFP or GAL4 at the endogenous translation initiation codon of DWnt2, DWnt4, DWnt5, DWnt6, DWnt10, and WntD, thus allowing the transcriptional activity to be readily assessed. DNA encoding an HA-tag was also inserted in the coding region of DWnt10 to generate a protein reporter (Figures S1B–S1D). Analysis of late third-instar wing imaginal discs showed that DWnt4 and DWnt6 are expressed, like Wingless, at the dorsal-ventral (DV) boundary (Figure 2A). DWnt2 was similarly expressed but more broadly. The expression of HA-DWnt10 was undetectable by anti-HA immunofluorescence. However, a weak GFP signal was produced in *DWnt10[GAL4] UAS-GFP* wing primordia, indicating a low-level expression (Figure S1E). The *DWnt5* and *WntD* reporters remained silent in wing primordia (Figures 1B and S1F). Since these Wnts do not bind the Fz CRD (Wu and Nusse, 2002), we conclude that they are unlikely to contribute to the activation of the core Fz PCP pathway. In contrast, the genes expressed in imaginal discs (DWnt2, DWnt4, DWnt6, DWnt10, and Wingless) continued to be expressed at pupal stages (Figures 1B and S1E), suggesting that any of them could play a role in PCP beyond the period of patterning and growth.

PCP Establishment in the Absence of a Wnt Gradient

To test if any Wnt gradient contributes to PCP in the developing wing, we sought to abrogate the activity of *DWnt2*, *DWnt4*, *DWnt6,* and *DWnt10* in the *wg*[*cNRT*] background. All these genes, except for *DWnt2*, are located within 100 kb of each other in the genome, excluding the possibility of recombining individual mutants (Figure S2A). We opted, therefore, for iterative rounds of CRISPR-Cas9-mediated gene targeting to generate multiple *DWnt* mutants in the *wg*[*cNRT*] background. First, we sequentially introduced indels at *DWnt6* and *DWnt10* on a chromosome carrying *wg*[*cNRT*]. Then we generated a conditional *DWnt4* allele (*DWnt4[cKO]*) in the background of *wg*[*cNRT*]*, DWnt6[KO],*and *DWnt10[KO]* to generate a quadruple mutant chromosome (*DWnt4[cKO], wg[cNRT], DWnt6[KO],* and *DWnt10[KO]*). A deletion of the first exon of *DWnt2*, which is located elsewhere on chromosome 2, was generated separately and introduced on this chromosome by standard recombination to generate a quintuple mutant chromosome (Figure S2B). Thus, with an *rn^gal4^*-driven Flp expression, we obtained a wing primordia expressing NRT-Wg instead of Wg and lacking DWnt2, DWnt4, DWnt6, and

CellPress
OPEN ACCESS

DWnt10, effectively removing all diffusible Wnts. The resulting fly wings were smaller than the wild type (Figure 2C), as previously reported for NRT-Wg wings (Alexandre et al., 2014) but, remarkably, wing hair orientation and the distribution of Stan were normal (Figure 2D). This finding suggests that a Wnt diffusion gradient is not necessary for the establishment and maintenance of the Fz-dependent PCP.

PCP without Secreted Wnt

In light of the previous result, we wondered whether Wnts (graded or otherwise) are at all required for the establishment of PCP in *Drosophila* wings. Since Wingless is required for wing specification and growth, complete and early removal of all Wnts leads to the absence of wing primordia, precluding an assessment of PCP. However, the core Fz PCP pathway is thought to be required only from early pupal stages, after most growth has taken place. Indeed, sensitive imaging techniques have shown that PCP domains start aligning along the proximal-distal axis from the late third-instar larval stage (Sagner et al., 2012; Aigouy et al., 2010). Moreover, the PCP phenotype of *fz* mutant could be rescued by the uniform Fz-GFP expression up until 6 h after prepupa formation (APF) (Strutt and Strutt, 2002). Therefore, it appears that the roles of Wnt signaling in growth and PCP can be temporally separated. We tested this further with a conditional allele of *dsh*, which is required for both activities (Figures S3A and S3B). Inactivation of this allele (*dsh*[*cKO*]) with *UAS-Flp* and nub^{gal4}, which is expressed specifically in wing primordia at the late second instar stage (Zirin and Mann, 2007) allowed sufficient growth to reveal the expected PCP phenotype (Figures S3C–S3E). There is, therefore, a temporal window when the role of Wnt ligands in PCP can be assessed independently of their role in growth.

All Wnts (except WntD) require the multi-pass transmembrane protein Wntless, aka Evenness interrupted, (here, referred to as Wls) for progression in the secretory pathway (Bänziger et al., 2006; Bartscherer et al., 2006; Herr and Basler, 2012). The complete loss of Wls effectively prevents the secretion of all Wnts, and the experimental abrogation of Wls could, therefore, be used to inhibit the activity of all Wnts at once. However, the Wls protein activity is known to perdure (Bänziger et al., 2006; Bartscherer et al., 2006), limiting the temporal resolution of a conditional allele or an RNAi-mediated interference. To overcome this limitation, we designed an approach to target the Wls protein as well as the gene. Inhibition of Wls protein was achieved by trapping it in the endoplasmic reticulum (ER), thereby preventing its progression, and that of all Wnts, through the secretory pathway. We first engineered the *wls* locus so that it expressed a functional GFP fusion, with the GFP moiety on the luminal side (*wls*[*ExGFP*]) (Figure S3F). We also created a transgene for the Gal4-dependent expression of an anti-GFP nanobody modified to be retained in the ER lumen (*UAS-Nanobody*^{KDEL}) (Figure 3A). Homozygous *wls*[*ExGFP*] larvae expressing this transgene under the control of vg^{gal4}, an early wing primordium driver, gave rise to flies lacking wings (Figure 3B), the same phenotype seen in *wingless*^{1} mutants, which lack the wing enhancer of *wg* (Sharma and Chopra, 1976). Hence, trapping Wls in the ER is an effective approach to inhibit Wnt secretion.

Having established the effectiveness of the Wls trapping approach, we turned to the nub^{gal4} driver to activate *UAS-Nanobody*^{KDEL} after sufficient growth has taken place. This was combined with a tissue-specific gene knockout technique involving the expression of *UAS-Cas9* in the presence of a transgene expressing a guide RNA targeting *wls* (Port and Bullock, 2016; Port et al., 2020). Thus, in the *wls*[*ExGFP*] background, one Gal4 driver suffices to induce expression of Cas9 (for gene inactivation) and Nanobody^{KDEL} (for sequestration of the protein product). In the resulting imaginal discs, Wls-GFP was no longer detectable at the cell surface (compare Figures 3C and 3E), confirming its trapping in the ER. Moreover, in the resulting pupal wing, Wingless was retained within Wingless-producing cells (compare Figures 3D and 3F), and therefore, unable to activate signal transduction. Indeed, adult wings of this genotype lacked margin tissue (Figure 3G), which is specified by canonical Wnt signaling (Couso et al., 1994; Micchelli et al., 1997). Occasional misoriented hairs were seen but only near areas of tissue deformation caused by the lack of margin (Figure S3G). Remarkably, hair orientation and Stan localization were normal in the central region of the wing (Figures 3G and 3H), far away from any Wnt sources at the margin, a strong indication that the global cue to PCP was not impaired. Therefore, PCP can be established in the absence of secreted Wnt ligands.

PCP without Signals from the Wing Margin

Our results show that Wnt ligands are not needed for the establishment of the core PCP pathway (Figure 4A). We next determined whether another signal originating from the prospective wing margin could serve as a global cue. Partial deletion of the margin, achieved with various mutant combinations has been achieved previously and shown not to affect PCP (Gubb and García-Bellido, 1982). We took advantage of improved genetic tools to ensure complete removal of the margin at a defined developmental time (Figure 4B). A combination of Flp, LexA, and Gal4-regulated transgenes were used to express Hid and Reaper, two pro-apoptotic proteins (Goyal et al., 2000) specifically in the prospective wing margin at the third instar stage (See details in Figures 4C–4I). Staining with anti-Wingless showed that most of the prospective margin was absent in third-instar wing discs (Figures 4C and 4G). Moreover, the resulting wing completely lacked recognizable margin tissues (compare Figures 1B′ and 4E). The wings were particularly small, probably because of a lack of Wingless signaling during the growth period (Figures 4E and 4F). Yet, PCP, as assayed by Stan staining in the pupal wing was normal (Figure 4I), suggesting that no signal emanating from the prospective margin is required for PCP.

DISCUSSION

There has been an ongoing debate whether Wnt ligands play a permissive or instructive role in PCP (Heisenberg et al., 2000; Ulrich et al., 2005; Witze et al., 2008; Gros et al., 2009; Gao et al., 2011, 2018; Wu et al., 2013; Chu and Sokol, 2016; Minegishi et al., 2017; Navajas Acedo et al., 2019). To assess the role of Wnt gradients rigorously, we engineered *Drosophila* larvae so that their wing primordia rely on a membrane-tethered Wingless as their only source of Wnt. This ensured that any diffusion-based Wnt gradient is eliminated, although, at the outset, a gradient based on cytonemes could not be excluded

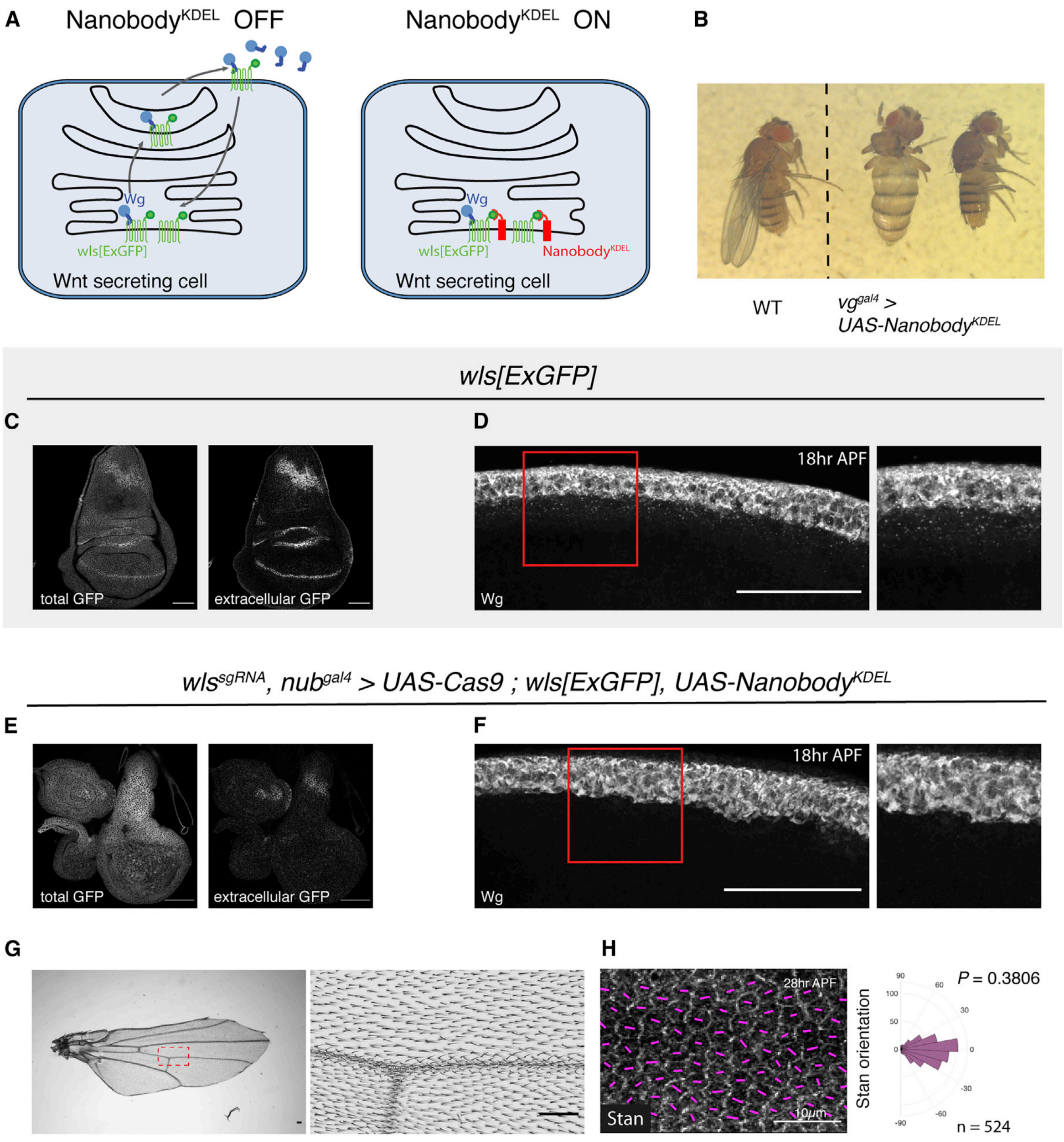

Figure 3. Inactivation of all DWnts during the Mid-Third Instar Does Not Impair PCP

(A) Diagram showing how Nanobody^{KDEL} is expected to prevent Wnt secretion.

(B) Expression of Nanobody^{KDEL} specifically in wing primordia and from the onset of development (with *vg*^{gal4}) in homozygous *wls*[*ExGFP*] larvae phenocopies a *wingless* mutant.

(C) Total and extracellular Wls[ExGFP] in a third-instar *wls*[*ExGFP*] wing disc. This recapitulates the pattern seen with wild-type Wls.

(D) anti-Wg staining in a *wls*[*ExGFP*] 18 h APF pupal wing showing the spread of Wingless, as indicated by the presence of internalized Wingless in cells flanking the wing margin.

(E) Wing disc showing the relative absence of Wls within the pouch (compared with the wild type in C).

(F) anti-Wg staining in a 18 h APF pupal wing, showing the lack of internalized Wingless away from the wing margin, confirming the absence of release from expressing cells (compare to D).

(G) Adult wing where all DWnts were inactivatedby driving the expression of Nanobody^{KDEL} and Cas9 with the wing pouch driver *nub*^{gal4}. This driver, which is located on the second chromosome was used instead of the previously used *rn*^{gal4} (third chromosome) to overcome constraints caused by the number of transgenic alleles already present on the 3rd chromosome.

(H) Stan polarization in pupal wings of the same genotype as (G). The p value was calculated using the two-sample Kolmogorov-Smirnov test to compare the distribution of Stan orientation of the mutant condition (H) to that of the wild type in Figure 1B″. Scale bars are 50 μm unless specified otherwise.

CellPress
OPEN ACCESS

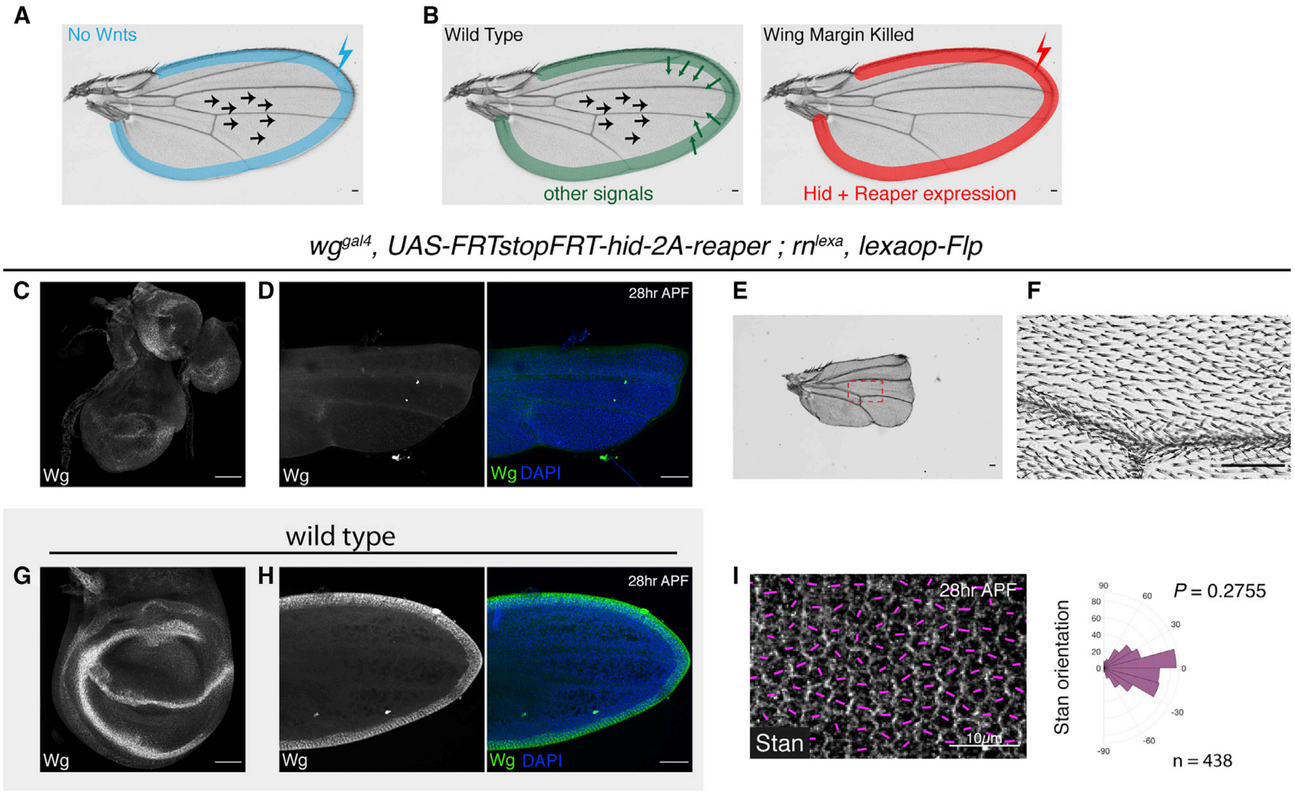

Figure 4. Margin Ablation during Early Third Instar Does Not Impair PCP

(A) Schematic illustrating that inactivation of all Wnts (expressed in the blue shaded area) is compatible with normal PCP in the wing.

(B) Experimental set-up to test whether another signal originating from the margin is required for PCP.

(C and D) Wingless staining in a third-instar wing disc and pupal wing with the prospective margin ablated. Outline of the pupal wing is apparent from DAPI staining (blue).

(E and F) Adult wing with margin ablated.

(G) Anti-Wg staining in the wild-type wing disc, as a control for (C). (H) Anti-Wg staining in the wild-type pupal wing, as a control for (D).

(I) Stan polarization in pupal wings with margin ablated. The p value was calculated using the two-sample Kolmogorov-Smirnov test to compare the distribution of Stan orientation of the mutant condition (I) to that of the wild type in Figure 1B″. Scale bars are 50 μm unless specified otherwise.

(Stanganello and Scholpp, 2016). To our surprise, wings entirely lacking diffusible Wnt had normal PCP, suggesting that a gradient of the Wnt ligand is not needed for Fz-dependent PCP, even if the localized ectopic Wnt can orient PCP both in *Drosophila* wing primordia (Wu et al., 2013) and in vertebrate tissues (Chu and Sokol, 2016; Minegishi et al., 2017). Therefore, we suggest that, while Wnt ligands can orient PCP in gain-of-function experiments, a Wnt gradient is not necessary. It is possible that ectopic Wnt can hijack the core PCP pathway in a non-physiological manner. Alternatively, a Wnt gradient could normally contribute to PCP but in a redundant manner with another global cue. Accordingly, in tissues where no such redundant system exists, a Wnt gradient might be essential, as suggested for the developing mouse limb (Gao et al., 2011). In any case, our results suggest that the global alignment of PCP could be achieved without a Wnt gradient.

Despite doubts about the instructive value of Wnt gradients in PCP, it has been generally accepted that, in vertebrates, Wnts are needed, at least in a permissive manner. Yet, as we have shown, in the wing primordia of *Drosophila*, PCP is established normally in the complete absence of secreted Wnt ligands (See also Ewen-Campen et al., 2020). This conclusion is based on two sets of experimental results. In one set, Wnt activity was prevented by trapping Wls, and hence all Wnts, in the ER. The effectiveness of this approach can be inferred from the observation that induction of trapping in early primordia completely prevented wing development. Using this approach to trap all Wnts at a later time, but before the period of PCP establishment, we found that PCP can be established normally in the absence of Wnt ligands. Occasional misoriented hairs were seen but only at the edge of the wing. We attribute these minor defects to local tissue deformation but cannot exclude the possibility they might arise because of the absence of Wnt. Our demonstration of global PCP establishment in the absence of Wnt is at odds with the established roles of Wnt5a and Wnt11 in the vertebrate mesoderm. Perhaps, these Wnt ligands have evolved a PCP role that is not present in *Drosophila*. This could, for example, be mediated by Ror2, a receptor tyrosine kinase that acts as a co-receptor for Wnt5a to establish PCP in the mouse limb bud (Gao et al., 2011). Our conclusion that Wnt ligands are dispensable for PCP in the *Drosophila* wing is further strengthened by our observation that PCP is unaffected by complete ablation of the

prospective margin, where all the relevant Wnts are produced. The result of our margin ablation experiment also shows that PCP can be established without another diffusible cue originating from the prospective margin.

If Wnts are not required for PCP, another global cue must exist. This cannot be from an entirely separate redundant system, since the removal of Fz1 on its own leads to strong PCP phenotypes. Therefore, any alternative global cue must feed into the Fz-dependent core pathway. Potential candidates include the ft/ds pathway and the mechanical forces associated with tissue morphogenesis, as summarized in recent reviews (Butler and Wallingford, 2017; Aw and Devenport, 2017). The coordination of PCP proteins' localization across an entire tissue is no mean feat and redundancy could help ensure robustness. Thus, multiple mechanisms would act together to establish PCP, with some cues having more influence than others depending on the developmental time and tissue context. Although a Wnt gradient could be important in some conditions, our results highlight a situation when Wnt secretion is entirely dispensable.

STAR★METHODS

Detailed methods are provided in the online version of this paper and include the following:

- KEY RESOURCES TABLE
- RESOURCE AVAILABILITY
  - Lead Contact
- MATERIALS AVAILABILITY
- DATA AND CODE AVAILABILITY
- EXPERIMENTAL MODEL AND SUBJECT DETAILS
  - *Drosophila* Strains and Fly Genetics
  - Genotypes
- METHOD DETAILS
  - Generation of *DWnt4[KO]* in *wg[cNRT]* Background
  - Generation of DWnt2, DWnt4, DWnt6, and DWnt5 GFP Reporters
  - Generation of *DWnt10[HA]* and *DWnt[GAL4]* Lines
  - Generation of Multiple DWnt Mutants in *wg[cNRT]* Background
  - Generation of *dsh[cKO]*
  - Generation of *UAS-Nanobody^{KDEL}*
  - Generation of *wls[ExGFP]*
  - Generation of *rn^{lexa}*
  - Immunostaining and Image Acquisition
- QUANTIFICATION AND STATISTICAL ANALYSIS
  - Polarity Measurement
  - Statistical Analysis

SUPPLEMENTAL INFORMATION

Supplemental Information can be found online at https://doi.org/10.1016/j.devcel.2020.08.004.

ACKNOWLEDGMENTS

We thank Emma Powell, Iris Salecker, and Fillip Port for providing fly lines and Joachim Kurth for *Drosophila* injections. This work was supported by core funding to the Francis Crick Institute (FC001204 to J.-P.V.), the Centre National de la Recherche Scientifique (CNRS) to Y.B., the European Union (ERC Advanced TiMorph 340784 to Y.B.) and ARC (SL220130607097 to Y.B.) J.J.S.Y. is supported by the Francis Crick Ph.D. Training Programme (FC001204 to J.-P.V.). A.M. is supported by Fondation ARC pour la Recherche sur le Cancer (PDF20180507510).

AUTHOR CONTRIBUTIONS

The project was conceived by J.J.S.Y., J.-P.V., and I.J.M.; the first draft of the paper was written by J.J.S.Y., J.-P.V., Y.B., and I.J.M.; all the authors contributed to the design and interpretation of experiments. Specific experimental contributions are as follows: generation of Wnt reporter lines (A.M.-C., S.P., Y.B., and J.J.S.Y.), generation of endogenously tagged Wls and conditional Dsh knockout lines (C.A. and I.J.M.), generation of individual and combinatorial Wnt mutant lines (J.J.S.Y.), PCP analysis (J.J.S.Y.).

DECLARATION OF INTERESTS

The authors declare no competing interests.

Received: May 21, 2020
Revised: July 7, 2020
Accepted: August 10, 2020
Published: September 14, 2020

REFERENCES

Adler, P.N. (2012). The frizzled/stan pathway and planar cell polarity in the Drosophila wing. Curr. Top. Dev. Biol. *101*, 1–31.

Adler, P.N., Krasnow, R.E., and Liu, J. (1997). Tissue polarity points from cells that have higher frizzled levels towards cells that have lower frizzled levels. Curr. Biol. *7*, 940–949.

Aigouy, B., Farhadifar, R., Staple, D.B., Sagner, A., Röper, J.C., Jülicher, F., and Eaton, S. (2010). Cell flow reorients the axis of planar polarity in the wing epithelium of Drosophila. Cell *142*, 773–786.

Alexandre, C., Baena-Lopez, A., and Vincent, J.P. (2014). Patterning and growth control by membrane-tethered wingless. Nature *505*, 180–185.

Andre, P., Song, H., Kim, W., Kispert, A., and Yang, Y. (2015). Wnt5a and Wnt11 regulate mammalian anterior-posterior axis elongation. Development *142*, 1516–1527.

Aw, W.Y., and Devenport, D. (2017). Planar cell polarity: global inputs establishing cellular asymmetry. Curr. Opin. Cell Biol. *44*, 110–116.

Axelrod, J.D. (2001). Unipolar membrane association of dishevelled mediates frizzled planar cell polarity signaling. Genes Dev *15*, 1182–1187.

Baena-Lopez, L.A., Alexandre, C., Mitchell, A., Pasakarnis, L., and Vincent, J.P. (2013). Accelerated homologous recombination and subsequent genome modification in Drosophila. Development *140*, 4818–4825.

Bänziger, C., Soldini, D., Schütt, C., Zipperlen, P., Hausmann, G., and Basler, K. (2006). Wntless, a conserved membrane protein dedicated to the secretion of Wnt proteins from signaling cells. Cell *125*, 509–522.

Bartscherer, K., Pelte, N., Ingelfinger, D., and Boutros, M. (2006). Secretion of Wnt ligands requires Evi, a conserved transmembrane protein. Cell *125*, 523–533.

Bastock, R., Strutt, H., and Strutt, D. (2003). Strabismus is asymmetrically localised and binds to Prickle and Dishevelled during Drosophila planar polarity patterning. Development *130*, 3007–3014.

Bhanot, P., Brink, M., Samos, C.H., Hsieh, J.C., Wang, Y., Macke, J.P., Andrew, D., Nathans, J., and Nusse, R. (1996). A new member of the frizzled family from Drosophila functions as a wingless receptor. Nature *382*, 225–230.

Bosch, P.S., Ziukaite, R., Alexandre, C., Basler, K., and Vincent, J.P. (2017). Dpp controls growth and patterning in Drosophila wing precursors through distinct modes of action. eLife *6*, e22546.

Butler, M.T., and Wallingford, J.B. (2017). Planar cell polarity in development and disease. Nat. Rev. Mol. Cell Biol. *18*, 375–388.

Casal, J., Lawrence, P.A., and Struhl, G. (2006). Two separate molecular systems, Dachsous/fat and starry night/frizzled, act independently to confer planar cell polarity. Development *133*, 4561–4572.

CellPress
OPEN ACCESS

Chae, J., Kim, M.J., Goo, J.H., Collier, S., Gubb, D., Charlton, J., Adler, P.N., and Park, W.J. (1999). The Drosophila tissue polarity gene starry night encodes a member of the protocadherin family. Development *126*, 5421–5429.

Chu, C.W., and Sokol, S.Y. (2016). Wnt proteins can direct planar cell polarity in vertebrate ectoderm. eLife *5*, e16463.

Clark, H.F., Brentrup, D., Schneitz, K., Bieber, A., Goodman, C., and Noll, M. (1995). Dachsous encodes a member of the cadherin superfamily that controls imaginal disc morphogenesis in Drosophila. Genes Dev *9*, 1530–1542.

Couso, J.P., Bishop, S.A., and Martinez Arias, A. (1994). The wingless signalling pathway and the patterning of the wing margin in Drosophila. Development *120*, 621–636.

Devenport, D., and Fuchs, E. (2008). Planar polarization in embryonic epidermis orchestrates global asymmetric morphogenesis of hair follicles. Nat. Cell Biol. *10*, 1257–1268.

Diao, F., Ironfield, H., Luan, H., Diao, F., Shropshire, W.C., Ewer, J., Marr, E., Potter, C.J., Landgraf, M., and White, B.H. (2015). Plug-and-play genetic access to Drosophila cell types using exchangeable exon cassettes. Cell Rep *10*, 1410–1421.

Ewen-Campen, B., Comyn, T., Vogt, E., and Perrimon, N. (2020). Wnt ligands are not required for planar cell polarity in the Drosophila wing or notum. bioRxiv biorxiv.org/content/10.1101/2020.06.05.137182v2.

Feiguin, F., Hannus, M., Mlodzik, M., and Eaton, S. (2001). The ankyrin repeat protein Diego mediates Frizzled-dependent planar polarization. Dev. Cell *1*, 93–101.

Fisher, K.H., and Strutt, D. (2019). A theoretical framework for planar polarity establishment through interpretation of graded cues by molecular bridges. Development *146*, dev168955.

Gao, B. (2012). Wnt regulation of planar cell polarity (PCP). Curr. Top. Dev. Biol. *101*, 263–295.

Gao, B., Ajima, R., Yang, W., Li, C., Song, H., Anderson, M.J., Liu, R.R., Lewandoski, M.B., Yamaguchi, T.P., and Yang, Y. (2018). Coordinated directional outgrowth and pattern formation by integration of Wnt5a and Fgf signaling in planar cell polarity. Development *145*, dev163824.

Gao, B., Song, H., Bishop, K., Elliot, G., Garrett, L., English, M.A., Andre, P., Robinson, J., Sood, R., Minami, Y., et al. (2011). Wnt signaling gradients establish planar cell polarity by inducing Vangl2 phosphorylation through Ror2. Dev. Cell *20*, 163–176.

Goodrich, L.V., and Strutt, D. (2011). Principles of planar polarity in animal development. Development *138*, 1877–1892.

Goyal, L., McCall, K., Agapite, J., Hartwieg, E., and Steller, H. (2000). Induction of apoptosis by Drosophila reaper, hid and grim through inhibition of IAP function. EMBO J *19*, 589–597.

Gratz, S.J., Ukken, F.P., Rubinstein, C.D., Thiede, G., Donohue, L.K., Cummings, A.M., and O'Connor-Giles, K.M. (2014). Highly specific and efficient CRISPR/Cas9-catalyzed homology-directed repair in Drosophila. Genetics *196*, 961–971.

Gros, J., Serralbo, O., and Marcelle, C. (2009). WNT11 acts as a directional cue to organize the elongation of early muscle fibres. Nature *457*, 589–593.

Gubb, D., and García-Bellido, A. (1982). A genetic analysis of the determination of cuticular polarity during development in Drosophila melanogaster. J. Embryol. Exp. Morphol. *68*, 37–57.

Hadjieconomou, D., Rotkopf, S., Alexandre, C., Bell, D.M., Dickson, B.J., and Salecker, I. (2011). Flybow: genetic multicolor cell labeling for neural circuit analysis in Drosophila melanogaster. Nat. Methods *8*, 260–266.

Heisenberg, C.P., Tada, M., Rauch, G.J., Saúde, L., Concha, M.L., Geisler, R., Stemple, D.L., Smith, J.C., and Wilson, S.W. (2000). Silberblick/Wnt11 mediates convergent extension movements during zebrafish gastrulation. Nature *405*, 76–81.

Heller, D., Hoppe, A., Restrepo, S., Gatti, L., Tournier, A.L., Tapon, N., Basler, K., and Mao, Y. (2016). EpiTools: an open-source image analysis toolkit for quantifying epithelial growth dynamics. Dev. Cell *36*, 103–116.

Herr, P., and Basler, K. (2012). Porcupine-mediated lipidation is required for Wnt recognition by Wls. Dev. Biol. *361*, 392–402.

Hsieh, J.C., Rattner, A., Smallwood, P.M., and Nathans, J. (1999). Biochemical characterization of Wnt-frizzled interactions using a soluble, biologically active vertebrate Wnt protein. Proc. Natl. Acad. Sci. USA *96*, 3546–3551.

Jenny, A., Darken, R.S., Wilson, P.A., and Mlodzik, M. (2003). Prickle and Strabismus form a functional complex to generate a correct axis during planar cell polarity signaling. EMBO J *22*, 4409–4420.

Jenny, A., Reynolds-Kenneally, J., Das, G., Burnett, M., and Mlodzik, M. (2005). Diego and Prickle regulate Frizzled planar cell polarity signalling by competing for Dishevelled binding. Nat. Cell Biol. *7*, 691–697.

Klingensmith, J., Nusse, R., and Perrimon, N. (1994). The Drosophila segment polarity gene dishevelled encodes a novel protein required for response to the wingless signal. Genes Dev *8*, 118–130.

Lawrence, P.A., and Casal, J. (2013). The mechanisms of planar cell polarity, growth and the Hippo pathway: some known unknowns. Dev. Biol. *377*, 1–8.

Lawrence, P.A., Casal, J., and Struhl, G. (2002). Towards a model of the organisation of planar polarity and pattern in the Drosophila abdomen. Development *129*, 2749–2760.

Lee, H.J., Shi, D.L., and Zheng, J.J. (2015). Conformational change of Dishevelled plays a key regulatory role in the Wnt signaling pathways. eLife *4*, e08142.

Lu, B., Usui, T., Uemura, T., Jan, L., and Jan, Y.N. (1999). Flamingo controls the planar polarity of sensory bristles and asymmetric division of sensory organ precursors in Drosophila. Curr. Biol. *9*, 1247–1250.

Lu, X., and Sipe, C.W. (2016). Developmental regulation of planar cell polarity and hair-bundle morphogenesis in auditory hair cells: lessons from human and mouse genetics. Wiley Interdiscip. Rev. Dev. Biol. *5*, 85–101.

Mahoney, P.A., Weber, U., Onofrechuk, P., Biessmann, H., Bryant, P.J., and Goodman, C.S. (1991). The fat tumor suppressor gene in Drosophila encodes a novel member of the cadherin gene superfamily. Cell *67*, 853–868.

Matis, M., and Axelrod, J.D. (2013). Regulation of PCP by the Fat signaling pathway. Genes Dev *27*, 2207–2220.

Micchelli, C.A., Rulifson, E.J., and Blair, S.S. (1997). The function and regulation of cut expression on the wing margin of Drosophila: Notch, Wingless and a dominant negative role for Delta and Serrate. Development *124*, 1485–1495.

Minegishi, K., Hashimoto, M., Ajima, R., Takaoka, K., Shinohara, K., Ikawa, Y., Nishimura, H., McMahon, A.P., Willert, K., Okada, Y., et al. (2017). A Wnt5 activity asymmetry and intercellular signaling via PCP proteins polarize node cells for left-right symmetry breaking. Dev. Cell *40*, 439–452.e4.

Navajas Acedo, J., Voas, M.G., Alexander, R., Woolley, T., Unruh, J.R., Li, H., Moens, C., and Piotrowski, T. (2019). PCP and Wnt pathway components act in parallel during zebrafish mechanosensory hair cell orientation. Nat. Commun. *10*, 3993.

Ng, M., Diaz-Benjumea, F.J., Vincent, J.P., Wu, J., and Cohen, S.M. (1996). Specification of the wing by localized expression of wingless protein. Nature *381*, 316–318.

Nusse, R. (2020). The Wnt homepage, 1997 – 2020. http://web.stanford.edu/group/nusselab/cgi-bin/wnt/.

Pinheiro, D., Hannezo, E., Herszterg, S., Bosveld, F., Gaugue, I., Balakireva, M., Wang, Z., Cristo, I., Rigaud, S.U., Markova, O., and Bellaïche, Y. (2017). Transmission of cytokinesis forces via E-cadherin dilution and actomyosin flows. Nature *545*, 103–107.

Port, F., and Bullock, S.L. (2016). Augmenting CRISPR applications in Drosophila with tRNA-flanked sgRNAs. Nat. Methods *13*, 852–854.

Port, F., Chen, H.M., Lee, T., and Bullock, S.L. (2014). Optimized CRISPR/Cas tools for efficient germline and somatic genome engineering in Drosophila. Proc. Natl. Acad. Sci. USA *111*, E2967–E2976.

Port, F., Strein, C., Stricker, M., Rauscher, B., Heigwer, F., Zhou, J., Beyersdörffer, C., Frei, J., Hess, A., Kern, K., et al. (2020). A large-scale resource for tissue-specific CRISPR mutagenesis in Drosophila. eLife *9*, e53865.

Rauch, G.J., Hammerschmidt, M., Blader, P., Schauerte, H.E., Strähle, U., Ingham, P.W., McMahon, A.P., and Haffter, P. (1997). Wnt5 is required for tail formation in the zebrafish embryo. Cold Spring Harb. Symp. Quant. Biol. *62*, 227–234.

Sagner, A., Merkel, M., Aigouy, B., Gaebel, J., Brankatschk, M., Jülicher, F., and Eaton, S. (2012). Establishment of global patterns of planar polarity during growth of the Drosophila wing epithelium. Curr. Biol. *22*, 1296–1301.

Sharma, R.P., and Chopra, V.L. (1976). Effect of the Wingless (wg1) mutation on wing and haltere development in Drosophila melanogaster. Dev. Biol. *48*, 461–465.

St Pierre, S.E., Galindo, M.I., Couso, J.P., and Thor, S. (2002). Control of Drosophila imaginal disc development by rotund and roughened eye: differentially expressed transcripts of the same gene encoding functionally distinct zinc finger proteins. Development *129*, 1273–1281.

Stanganello, E., and Scholpp, S. (2016). Role of cytonemes in Wnt transport. J. Cell Sci. *129*, 665–672.

Struhl, G., Casal, J., and Lawrence, P.A. (2012). Dissecting the molecular bridges that mediate the function of Frizzled in planar cell polarity. Development *139*, 3665–3674.

Strutt, D.I. (2001). Asymmetric localization of frizzled and the establishment of cell polarity in the Drosophila wing. Mol. Cell *7*, 367–375.

Strutt, H., Gamage, J., and Strutt, D. (2016). Robust asymmetric localization of planar polarity proteins is associated with organization into signalosome-like domains of variable stoichiometry. Cell Rep *17*, 2660–2671.

Strutt, H., and Strutt, D. (2002). Nonautonomous planar polarity patterning in Drosophila: dishevelled-independent functions of frizzled. Dev. Cell *3*, 851–863.

Strutt, H., Warrington, S.J., and Strutt, D. (2011). Dynamics of core planar polarity protein turnover and stable assembly into discrete membrane subdomains. Dev. Cell *20*, 511–525.

Tada, M., and Smith, J.C. (2000). Xwnt11 is a target of Xenopus brachyury: regulation of gastrulation movements via Dishevelled, but not through the canonical Wnt pathway. Development *127*, 2227–2238.

Taylor, J., Abramova, N., Charlton, J., and Adler, P.N. (1998). Van Gogh: a new Drosophila tissue polarity gene. Genetics *150*, 199–210.

Thomas, C., and Strutt, D. (2012). The roles of the cadherins Fat and Dachsous in planar polarity specification in Drosophila. Dev. Dyn. *241*, 27–39.

Tree, D.R., Shulman, J.M., Rousset, R., Scott, M.P., Gubb, D., and Axelrod, J.D. (2002). Prickle mediates feedback amplification to generate asymmetric planar cell polarity signaling. Cell *109*, 371–381.

Ulrich, F., Krieg, M., Schötz, E.M., Link, V., Castanon, I., Schnabel, V., Taubenberger, A., Mueller, D., Puech, P.H., and Heisenberg, C.P. (2005). Wnt11 functions in gastrulation by controlling cell cohesion through Rab5c and E-cadherin. Dev. Cell *9*, 555–564.

Usui, T., Shima, Y., Shimada, Y., Hirano, S., Burgess, R.W., Schwarz, T.L., Takeichi, M., and Uemura, T. (1999). Flamingo, a seven-pass transmembrane cadherin, regulates planar cell polarity under the control of Frizzled. Cell *98*, 585–595.

VanderVorst, K., Hatakeyama, J., Berg, A., Lee, H., and Carraway, K.L. (2018). Cellular and molecular mechanisms underlying planar cell polarity pathway contributions to cancer malignancy. Semin. Cell Dev. Biol. *81*, 78–87.

Venken, K.J., Schulze, K.L., Haelterman, N.A., Pan, H., He, Y., Evans-Holm, M., Carlson, J.W., Levis, R.W., Spradling, A.C., Hoskins, R.A., and Bellen, H.J. (2011). MiMIC: a highly versatile transposon insertion resource for engineering Drosophila melanogaster genes. Nat. Methods *8*, 737–743.

Vinson, C.R., Conover, S., and Adler, P.N. (1989). A Drosophila tissue polarity locus encodes a protein containing seven potential transmembrane domains. Nature *338*, 263–264.

Wallingford, J.B., Rowning, B.A., Vogeli, K.M., Rothbächer, U., Fraser, S.E., and Harland, R.M. (2000). Dishevelled controls cell polarity during Xenopus gastrulation. Nature *405*, 81–85.

Wallingford, J.B., Vogeli, K.M., and Harland, R.M. (2001). Regulation of convergent extension in Xenopus by Wnt5a and Frizzled-8 is independent of the canonical Wnt pathway. Int. J. Dev. Biol. *45*, 225–227.

Witze, E.S., Litman, E.S., Argast, G.M., Moon, R.T., and Ahn, N.G. (2008). Wnt5a control of cell polarity and directional movement by polarized redistribution of adhesion receptors. Science *320*, 365–369.

Wolff, T., and Rubin, G.M. (1998). Strabismus, a novel gene that regulates tissue polarity and cell fate decisions in Drosophila. Development *125*, 1149–1159.

Wong, L.L., and Adler, P.N. (1993). Tissue polarity genes of Drosophila regulate the subcellular location for prehair initiation in pupal wing cells. J. Cell Biol. *123*, 209–221.

Wu, C.H., and Nusse, R. (2002). Ligand receptor interactions in the Wnt signaling pathway in Drosophila. J. Biol. Chem. *277*, 41762–41769.

Wu, J., Roman, A.C., Carvajal-Gonzalez, J.M., and Mlodzik, M. (2013). Wg and Wnt4 provide long-range directional input to planar cell polarity orientation in Drosophila. Nat. Cell Biol. *15*, 1045–1055.

Yang, W., Garrett, L., Feng, D., Elliott, G., Liu, X., Wang, N., Wong, Y.M., Choi, N.T., Yang, Y., and Gao, B. (2017). Wnt-induced Vangl2 phosphorylation is dose-dependently required for planar cell polarity in mammalian development. Cell Res *27*, 1466–1484.

Yang, Y., and Mlodzik, M. (2015). Wnt-Frizzled/planar cell polarity signaling: cellular orientation by facing the wind (Wnt). Annu. Rev. Cell Dev. Biol. *31*, 623–646.

Yates, L.L., Papakrivopoulou, J., Long, D.A., Goggolidou, P., Connolly, J.O., Woolf, A.S., and Dean, C.H. (2010). The planar cell polarity gene Vangl2 is required for mammalian kidney-branching morphogenesis and glomerular maturation. Hum. Mol. Genet. *19*, 4663–4676.

Zeidler, M.P., Perrimon, N., and Strutt, D.I. (1999). The four-jointed gene is required in the Drosophila eye for ommatidial polarity specification. Curr. Biol. *9*, 1363–1372.

Zirin, J.D., and Mann, R.S. (2007). Nubbin and Teashirt mark barriers to clonal growth along the proximal-distal axis of the Drosophila wing. Dev. Biol. *304*, 745–758.

STAR★METHODS

KEY RESOURCES TABLE

REAGENT or RESOURCE	SOURCE	IDENTIFIER
**Antibodies**		
mouse anti-Stan	DSHB	Cat# Flamingo #74 RRID:AB_528247
rat anti-shg	DSHB	Cat# DCAD2 RRID:AB_528120
mouse anti-Wg	DSHB	Cat# 4D4 RRID: AB_528512
rabbit anti-GFP	Abcam	Cat# ab6556 RRID:AB_305564
Alexa Fluor 488 Goat anti-Rat IgG (H+L) Cross-adsorbed Secondary Antibody	Thermo Fisher Scientific	Cat# A-11006 RRID:AB_2534074
Alexa Fluor 488 Goat anti-Mouse IgG (H+L)	Thermo Fisher Scientific	Cat# A28175 RRID:AB_2536161
Alexa Fluor 555 Plus Goat anti-Mouse IgG (H+L) Highly Cross-adsorbed Secondary Antibody	Thermo Fisher Scientific	Cat# A32727 RRID:AB_2633276
Alexa Fluor 647 Goat anti-Rat IgG (H+L) Cross-adsorbed Secondary Antibody	Thermo Fisher Scientific	Cat# A-21247 RRID:AB_141778
Alexa Fluor 647 Donkey anti-Rabbit IgG H&L	Abcam	Cat# ab150075 RRID:AB_2752244
**Chemicals, Peptides, and Recombinant Proteins**		
16% formaldehyde, methanol free	Thermo Fisher Scientific	Cat# 28906
Vectashield with DAPI	Vector Laboratories	Cat# H-1200 RRID:AB_2336790
**Experimental Models: Organisms/Strains**		
wg[cNRT]	Alexandre et al., 2014	N/A
DWnt4KO, wg[cNRT]	This paper	N/A
rn^{gal4}	Bloomington Drosophila Stock Center	RRID:BDSC_7405 Flybase: FBal0137099
UAS-Flp(III)	Bloomington Drosophila Stock Center	RRID:BDSC_4540 Flybase: FBst0004540
gfp-wg	Port et al., 2014	N/A
nlsGFP-DWnt2	This paper	N/A
nlsGFP-DWnt4	This paper	N/A
nlsGFP-DWnt5	This paper	N/A
nlsGFP-DWnt6	This paper	N/A
Dwnt10[HA]	This paper	N/A
Dwnt10[GAL4]	This paper	N/A
DWntD[GAL4]	This paper	N/A
Dwnt2KO, Dwnt4[cKO], wg[cNRT], Dwnt6[KO], Dwnt10[KO]	This paper	N/A
UAS-Flp5	lab of Iris Salecker	N/A
$Fz1^{P21}$	Bloomington Drosophila Stock Center	RRID:BDSC_41787 Flybase: FBal0004937
vg^{gal4}	This paper	N/A
$UAS\text{-}Nanobody^{KDEL}$	This paper	N/A
wls[ExGFP]	This paper	N/A
nub^{gal4}	Bloomington Drosophila Stock Center	RRID:BDSC_25754 Flybase: FBti0016825

(*Continued on next page*)

Continued

REAGENT or RESOURCE	SOURCE	IDENTIFIER
UAS-cas9	Port et al., 2020	N/A
wls^{sgRNA}	Port et al., 2020	N/A
wg^{gal4}	Alexandre et al., 2014	N/A
rn^{lexa}	This paper	N/A
lexaop-flp	lab of Iris Salecker	N/A
UAS-FRT stop FRT-hid-2A-reaper	lab of Iris Salecker	N/A
dsh[cKO]	This paper	N/A
Oligonucleotides		
Table S1	This work	N/A
Software and Algorithms		
Fiji	https://fiji.sc/	N/A
GraphPad Prism	GraphPad Software, Inc.	N/A
MATLAB_R2014b	Mathworks	N/A
Tissue Analyzer	Aigouy et al., 2010 https://grr.gred-clermont.fr/labmirouse/software/WebPA/	N/A
Polarity measurement MATLAB scripts	Strutt et al., 2016	N/A

RESOURCE AVAILABILITY

Lead Contact

Further information and requests for resources and reagents should be directed to and will be fulfilled by the Lead Contact, Jean-Paul Vincent (jp.vincent@crick.ac.uk).

MATERIALS AVAILABILITY

Fly lines generated in this study are available upon request.

DATA AND CODE AVAILABILITY

This study did not generate new datasets or codes.

EXPERIMENTAL MODEL AND SUBJECT DETAILS

Drosophila Strains and Fly Genetics

Fly strains were raised on standard agar media at 25°C, unless stated otherwise. Strains used in this paper were summarized in the Key Resources Table. DNA injection was performed by either BestGene or the Crick fly facility.

The Wnt reporter toolbox was generated for this study: nlsGFP reporters for DWnt2, DWnt4, DWnt6, and DWnt5; *DWnt10[HA]*; *DWnt10[GAL4]*; *DWntD[GAL4]*. Other fly lines generated in this study include: *DWnt4[KO], wg[cNRT]*; *DWnt2[KO], DWnt4[cKO], wg[cNRT], DWnt6-KO], DWnt10[KO]*; *fz1*^{P21}; *UAS-Nanobody*^{KDEL}; *wls[ExGFP]*; *dsh[cKO]*; *rn*^{lexa}. *wls*^{sgRNA} and *UAS-Cas9* were gifts from Fillip Port (Port et al., 2020). *UAS-FRT stop FRT-hid-2A-reaper*, *lexaop-Flp*, and *UAS-Flp5* were gifts from Iris Salecker. *wg*^{gal4} used in this study was as described in (Alexandre et al., 2014). *wg::GFP* was a gift from Simon Bullock (Port et al., 2014). The following stocks were obtained from the Bloomington Drosophila Stock Centre: *rn*^{gal4}; *UAS-Flp*; *nub*^{gal4}; *UAS-GFP*.

Genotypes

Figure 1

(B) *w1118*
(C) *wg[cNRT]; rn*^{gal4}*, UAS-Flp*
(D) *DWnt4[KO], wg[cNRT]; rn*^{gal4}*, UAS-Flp*
(E) *fz1*^{P21}

Figure 2

(A-B) *wg::GFP*
nls-GFP-DWnt (for DWnt2, DWnt4, DWnt5, and DWnt6)
DWnt10[HA]

(C-D) *DWnt2[KO], DWnt4[cKO], wg[cNRT], DWnt6[KO], DWnt10[KO]; rn^{gal4}, UAS-Flp, UAS-Flp5*

Figure 3

(B) *vg^{gal4}, UAS-$Nanobody^{KDEL}$*

(C-D) *wls[ExGFP]*

(E-H) *wls^{sgRNA}, nub^{gal4}, UAS-Cas9; wls[ExGFP], UAS-$Nanobody^{KDEL}$*

Figure 4

(C-F, I) *wg^{gal4}, UAS-FRT stop FRT-hid-2A-reaper; rn^{lexa}, lexaop-Flp*

(G-H) *w1118*

Figure S1

(E) *DWnt10[GAL4]/+; UAS-GFP/+*

(F) *DWntD[GAL4]/UAS-GFP*

Figure S3

(D-E) *dsh[cKO]/Y; nub^{gal4}, UAS-Flp*

(G) *wls^{sgRNA}, nub^{gal4}, UAS-Cas9; wls[ExGFP], UAS-$Nanobody^{KDEL}$*

METHOD DETAILS

Generation of *DWnt4[KO]* in *wg[cNRT]* Background

The *wg[cNRT]* conditional allele *(FRT wg FRT nrt-wg)* was generated by replacing the endogenous *wg* locus with *FRT wg FRT nrt-wg*, as described in (Alexandre et al., 2014). The *DWnt4[KO], wg[cNRT]* chromosome was generated via CRISPR-Cas9 and homologous recombination mediated repair in the *wg[cNRT]* background. The first exon of *DWnt4* was replaced with *attP* and *pax-GFP*, a selection marker, using the pTV^{GFP} targeting vector with 1.2kb 5' and 1.5kb 3' homology arms (Figure S1A). CRISPR target sites were chosen in unconserved regions, one upstream of the 5'UTR (ATGAGCAAAATGCAATCTAT), one in the intronic region following exon 1 (AGCATTTGAGGACGGCAAAC). The resulting pTV^{GFP}-*DWnt4[KO]* construct and the *$DWn4^{sgRNA}$* donor vector were co-injected into embryos from a cross of *nanos-Cas9* line and *wg[cNRT]*. Successful transformants were identified by GFP expression in the eyes, and subsequently, PCR verified.

Generation of DWnt2, DWnt4, DWnt6, and DWnt5 GFP Reporters

To generate the nls-GFP reporters of DWnt2, DWnt4, DWnt6 and DWnt5 expression, a nls-GFP-T2A targeting vector was built by introducing an nls encoding sequence (CCTAAGAAGAAGCGGAAAGTA) and a T2A sequence (GAGGGCCGCGGCTCCCTGCT GACCTGCGGCGACGTGGAGGAGAACCCCGGCCCC) upstream and downstream of GFP sequence, respectively in the $CHE929^{GFP\text{-}lox-mini\text{-}white\text{-}lox}$ vector, which allows transformant selection with *mini-white* (Pinheiro *et al.*, 2017). At least 1kb of 5' and 3' homology arms were cloned using the primers in Table S1.

Target sites were chosen using http://targetfinder.flycrispr.neuro.brown.edu/ website (Gratz *et al.*, 2014) to avoid off-target sites. sgRNAs were cloned in *pCFD5: U6:3-t::gRNA* vector (Port *et al.*, 2014). For all the reporters, the sgRNA vector and the *nls-GFP-T2A* targeting vector were injected into vas-Cas9 lines (Gratz *et al.*, 2014) by Bestgene, and subsequently, PCR verified.

Generation of *DWnt10[HA]* and *DWnt[GAL4]* Lines

The *DWnt10[HA]* allele was generated via two rounds of modifications (Figure S1C). In the first step, the first exon of *DWnt10* was replaced with *attP* and *pax-Cherry*, using the pTV^{Cherry} targeting vector with 1kb 5' and 1.5kb 3' homology arms. Target sites were chosen in unconserved regions, one upstream of the 5'UTR (TGCTTTAAATACAAGAATGC), one in the intronic region following exon 1 (TGAGATAAGAAGATGTTCAG). The resulting pTV^{Cherry}-*$DWnt10[KO]^{attP}$* and *$DWnt10^{sgRNA}$* vectors were co-injected into embryos from the *nanos-Cas9* line. Successful candidates were identified by Cherry expression in the eyes and subsequently, PCR verified. This created a null allele of *DWnt10* (*$DWnt10[KO]^{attP}$*). The attP site was then used for the reintegration of RIV^{white} (Baena-Lopez *et al.*, 2013) modified as follows. A DNA fragment containing the 5′UTR, CDS, and 3′UTR of DWnt10 was synthesized by GeneWiz, with the sequence of HA-tag inserted in an unconserved region in exon 6. This fragment was cloned into the RIV^{white} vector. The resulting RIV^{white}-*DWnt10-HA* vector was then injected into the *$DWnt10[KO]^{attP}$* line to generate *DWnt10[HA]* via PhiC31-mediated integration.

DWnt10[GAL4] was generated using the same strategy as for *DWnt10[HA]*, but instead of the RIV^{white} integration vector, a RIV^{gal4} integration vector (Baena-Lopez *et al.*, 2013) was inserted into the *attP* site of *$DWnt10[KO]^{attP}$* (Figure S1D).

DWntD[GAL4] was generated via two rounds of modification (Figure S1D). First, exon 1 was replaced with *attP* and *pax-Cherry*, using the pTV^{Cherry} targeting vector with 1.5kb 5' and 1.5kb 3' homology arms. *$DWntD^{sgRNA}$* was designed to target sites in unconserved regions, one upstream of the 5'UTR (GCTATATAAGTGTGCTGACC), one downstream of the 3'UTR (GTTTTAGCTACA GGTGGTTT). The pTV^{Cherry}-*$DWntD[KO]^{attP}$* and *$DWntD^{sgRNA}$* plasmids were co-injected into embryos from the *nanos-Cas9* line to generate the null allele *$DWntD[KO]^{attP}$* (PCR verified). A RIV^{gal4} integration vector (Baena-Lopez *et al.*, 2013) was then introduced by PhiC31-mediated integration to generate *DWntD[GAL4]* (Figure S1D).

Generation of Multiple DWnt Mutants in *wg[cNRT]* Background

First, a double knockout of *DWnt6 and DWnt10* was sequentially generated on the *wg[cNRT]* chromosome by injecting sgRNAs targeting the first exon of both genes in *wg[cNRT] nos-Cas9* embryos. Details of the target sites and cloning strategies are described in Figure S2B. Indels were screened by genomic DNA extraction and PCR sequencing of homozygous candidates.

Next, a conditional *DWnt4* allele, *DWnt4[cKO]*, was generated by CRISPR-Cas9 and homologous recombination-mediated repair on the *wg[cNRT], DWnt6[KO], DWnt10[KO]* chromosome. *DWnt4* was made conditional by replacing the 5′UTR and exon 1 with the same sequence but flanked by FRT71 sites (Figure S2B). CRISPR target sites were chosen in unconserved regions, one upstream of the 5'UTR (ATGAGCAAAATGCAATCTAT), one in the intronic region following exon 1 (AGCATTTGAGGACGGCAAAC). The rescuing pTV^{GFP}-*DWnt4[cKO]* construct was made by first generating PCR fragments encoding the 5′ arm and exon 1 of *DWnt4*. They were then stitched together and inserted into pTV^{GFP} upstream of the pax-GFP selection cassette by Gibson Assembly. FRT71 sites were included in the primers so that they would be inserted between the 5'arm and the rescuing exon 1 and also immediately after the rescuing exon 1. The 3' arm was then amplified by PCR and inserted after the pax-GFP selection cassette. The resulting pTV^{GFP}-*DWnt4[cKO]* plasmid was co-injected with *DWn4*^{sgRNA} into embryos from a cross of *nanos-Cas9* line and the *wg[cNRT], DWnt6[KO], DWnt10[KO]* line. Successful candidates were identified by pax-GFP expression and subsequently, PCR verified.

A *DWnt2[KO]* was generated separately by replacing the first exon with *attP* and *pax-Cherry*, using the pTV^{Cherry} targeting vector with 1.5kb 5' and 1.5kb 3' homology arms (Figure S2B). CRISPR target sites were chosen in the unconserved regions, one upstream of the 5′UTR (AGTAGTAGTACTACTTGATC), one in the intronic region following exon 1 (AAATCAAAATACCTTCATCG). The resulting pTV^{Cherry}-*DWnt2[KO]*^{attP} plasmid was co-injected with *DWnt2*^{sgRNA} into *nanos-Cas9* embryos. Successful candidates were identified by pax-Cherry expression and subsequently, PCR verified.

The *DWnt2[KO]* generated was then recombined with *DWnt4[cKO], wg[cNRT], DWnt6[KO], DWnt10[KO]*. Successful recombination was screened by the presence of pax-GFP (from *DWnt4[cKO]*), and extra bright pax-Cherry signal in the eye, as both the *DWnt2 [KO]* and *wg[cNRT]* alleles harbor the pax-Cherry marker. The recombinant was subsequently verified via PCR.

Generation of *dsh[cKO]*

The *dsh[cKO]* allele was generated in two steps (Figure S3B). First, pTV^{Cherry} with a 2kb 5'arm and 1kb 3'arm was used to replace the coding region with an attP site and pax-Cherry to generate *dsh[KO]*^{attP}. CRISPR target sites were chosen in the 5'UTR and just after the stop codon (TTCCCGTGGATTTCCGCAGT, CGCAGTCGGCGCAGCTAAAA, CTACAATACGTAATTAAATA, and TACGGA TACGTCCTGATCGT). The resulting pTV^{Cherry}-*dsh[KO]*^{attP} plasmid was co-injected with *dsh*^{sgRNA} into *nanos-Cas9* embryos. Successful candidates were identified by pax-Cherry expression and subsequently, PCR verified. This created a null allele of *dsh* (*dsh[KO]*^{attP}). The attP site was then used for the reintegration of *dsh-GFP* flanked by FRT sites using RIV10$^{dsh\text{-}GFP}$.

RIV10$^{dsh\text{-}GFP}$ was generated by first cloning *dsh* from genomic DNA into *pBS-KS*. The pBS-KS^{dsh} vector was then opened using a unique SnaBI site just prior to the stop codon of dsh. GFP was amplified via PCR from pEGFP-N1 (Clontech) and inserted into the linearized pBS-KS^{dsh} vector using Gibson Assembly to generate pBS-KS$^{dsh\text{-}GFP}$. Dsh contains a 'YVL' PDZ motif at the carboxy-terminus that has been suggested to be essential for function (Lee *et al.*, 2015). This 'YVL' motif was hence duplicated and inserted after the GFP coding sequence. The *dsh-GFP* sequence was then subcloned from the pBS-KS$^{dsh\text{-}GFP}$ vector into RIV10$^{attB\text{-}paxGFP}$ via the NheI and AgeI restriction sites to generate RIV10$^{dsh\text{-}GFP}$. The RIV10$^{dsh\text{-}GFP}$ vector was then injected into the *dsh*[*KO*]^{attP} line to generate the *dsh*[*cKO*] line via PhiC31-mediated integration.

Generation of *UAS-Nanobody*^{KDEL}

UAS-Nanobody^{KDEL} was generated by PCR amplifying the coding region of the VHH4 nanobody from *pHT201* (gift from Dr. Peter Thorpe, Queen Mary University of London), and subcloning it into *pUAST*. The sequence encoding KDEL was contained within the reverse primer used for amplification of the nanobody, such that the KDEL was located at the C-terminal end of the nanobody just prior to the stop codon. pUAST-*Nanobody*^{KDEL} was then randomly integrated via P-element insertion and one line on the third chromosome was recovered.

Generation of *wls[ExGFP]*

The *wls[ExGFP]* line was generated by two rounds of injection (Figure S3F). First, using the accelerated 'Ends out' homologous recombination method described in (Baena-Lopez *et al.*, 2013), a region comprising the three exons of *wls* (from 20bp upstream of the initiation codon till 49bp after the stop codon) was replaced by an attP site and pax-Cherry, using the pTV^{Cherry} integration vector, to generate *wls[KO]*^{attP}. In a second step, a DNA fragment encoding *wls[ExGFP]* was generated starting with Lit28-EVI^{2XHA}, which was generated as follows. DNA encoding Evi2XHA was synthesized by Genewiz, such that two HA tags flanked by Gly/Ala linker were inserted between amino acid 506(D) and 507(N). The native 5'UTR and the 3'UTR of *wls* were subsequently added to generate an evi2XHA cDNA. The resulting Lit28-EVI^{2XHA} was digested with AatII. This allowed the HA tags to be replaced with GFP (amplified with primers including the AatII sites from pEGFP-N1 (Clontech)). DNA encoding Wls[ExGFP] was then sub-cloned from Lit28 into RIV^{Cherry}, which was subsequently injected into the *wls[KO]*^{attP} to generate the *wls[ExGFP]* line via PhiC31-mediated integration.

Generation of *rn^lexa*

Rn-LexA was generated using RMCE (Venken *et al.*, 2011), to insert *pBS-KS-attB2-SA-T2A-LexA::GADfluw-Hsp70* into a *rn* MIMIC line (Bloomington, #44158). *pBS-KS-attB2-SA-T2A-LexA::GADfluw-Hsp70* was a gift from Benjamin White (Addgene plasmid # 78304). (Diao *et al.*, 2015)

Immunostaining and Image Acquisition

The primary antibodies used were: mouse anti-Stan (1:10, pre-adsorbed, DSHB Flamingo #74), rat anti-Shg (1:50, pre-adsorbed, DSHB DCAD2); mouse anti-Wg (1:500, DSHB 4D4), rabbit anti-GFP (1:500, Abcam ab6556). Secondary antibodies (Alexa Fluor 488, 555, 647) were used at 1:200.

Larval wing discs and pupal wings were dissected and fixed in PBS 4% formaldehyde for 20min (larval discs) or 1 h (pupal wings). Dissected tissues were then washed in 0.1% PBT three times and then incubated in a blocking solution (0.1% BSA) for 1hr. Samples were incubated in primary antibodies overnight at 4°C, and then washed in PBT three times. Secondary antibodies in a blocking solution were then added and incubated for 2 h at room temperature. Samples were then washed three times in PBT, and then mounted in Vectashield with DAPI. All immunofluorescence images were acquired from a Leica SP5 confocal microscope. Adult wings images were obtained from a wide field microscope (Zeiss Axiovert 200M).

QUANTIFICATION AND STATISTICAL ANALYSIS

Polarity Measurement

Z-stacks of the acquired images of each wing were projected using a MATLAB script, modified from (Heller et al., 2016), which allowed the projection of the apical regions of epithelial tissues into 2D images, based on the anti-shg immunofluorescent staining. Membrane masks were generated using Tissue Analyzer (Aigouy et al., 2010). To determine the asymmetric localization of Stan of individual cells, a MATLAB script was used (Strutt et al., 2016), based on Stan staining intensity in the pupal wing. This script also generated PCP nematics for each individual cell, which takes into account the orientation and magnitude of the Stan polarization. This nematic order was visualized as magenta lines superimposed onto the original anti-Stan immunofluorescence image. Polar histograms were generated in MATLAB to visualize the orientation of Stan, such that 0° was oriented as pointing distally in the pupal and adult wings.

Statistical Analysis

The two sample Kolmogorov-Smirnov test was used as a statistical test to compare the difference in the distributions of the cellular polarity of cells from two independent samples that contain non-independent data, such as the case in PCP analyses when comparing wild type versus mutant conditions.

Developmental Cell, Volume *54*

Supplemental Information

Frizzled-Dependent Planar Cell Polarity

without Secreted Wnt Ligands

Joyce J.S. Yu, Aude Maugarny-Calès, Stéphane Pelletier, Cyrille Alexandre, Yohanns Bellaiche, Jean-Paul Vincent, and Ian J. McGough

Supplementary Figure 1. Reporters of DWnt expression (Related to main Figure 1D and 2A-B)

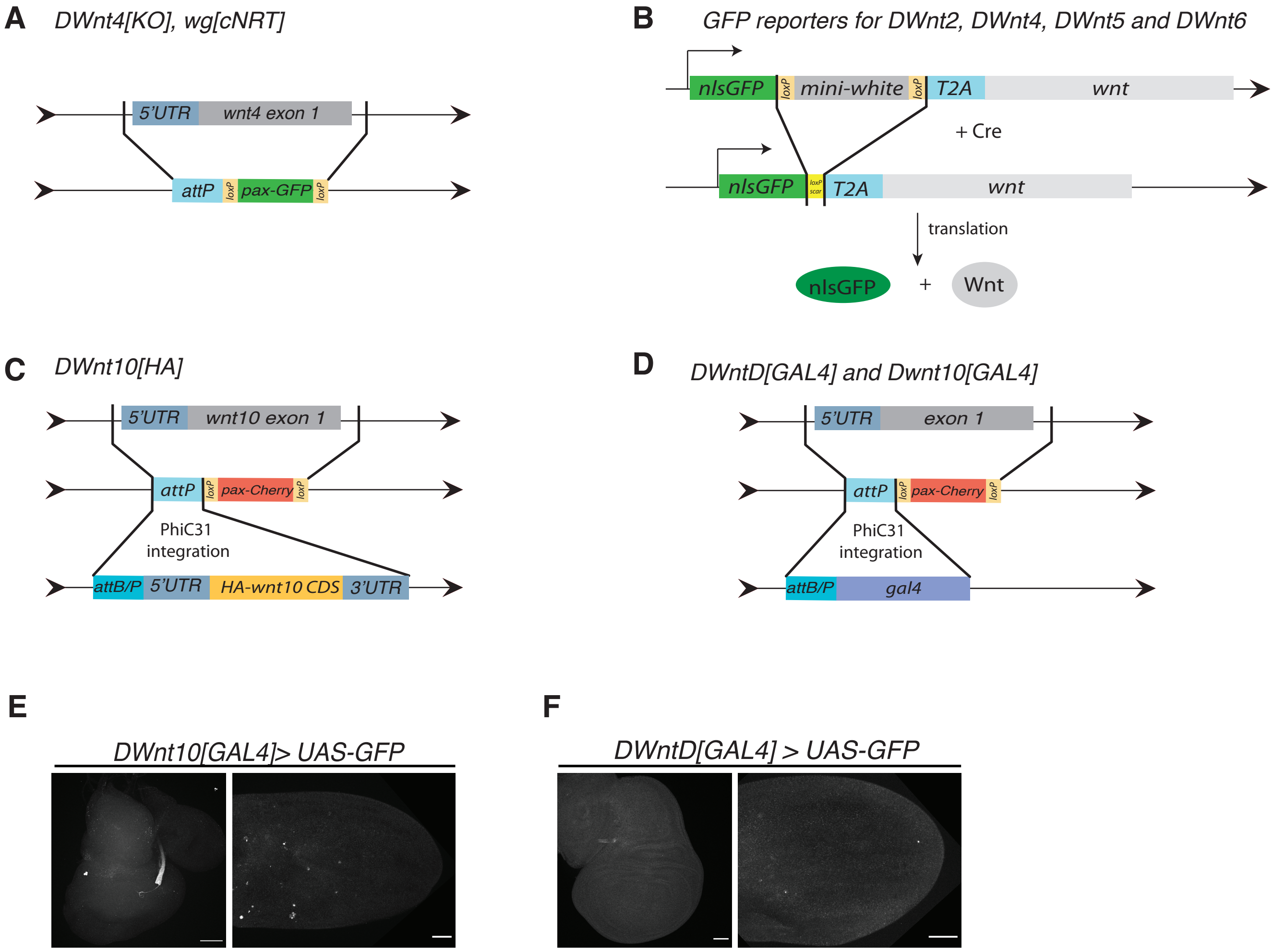

Supplementary Figure 2. A strain for the manipulation of five DWnt genes simunltaneously (Related to main Figure 2C-D)

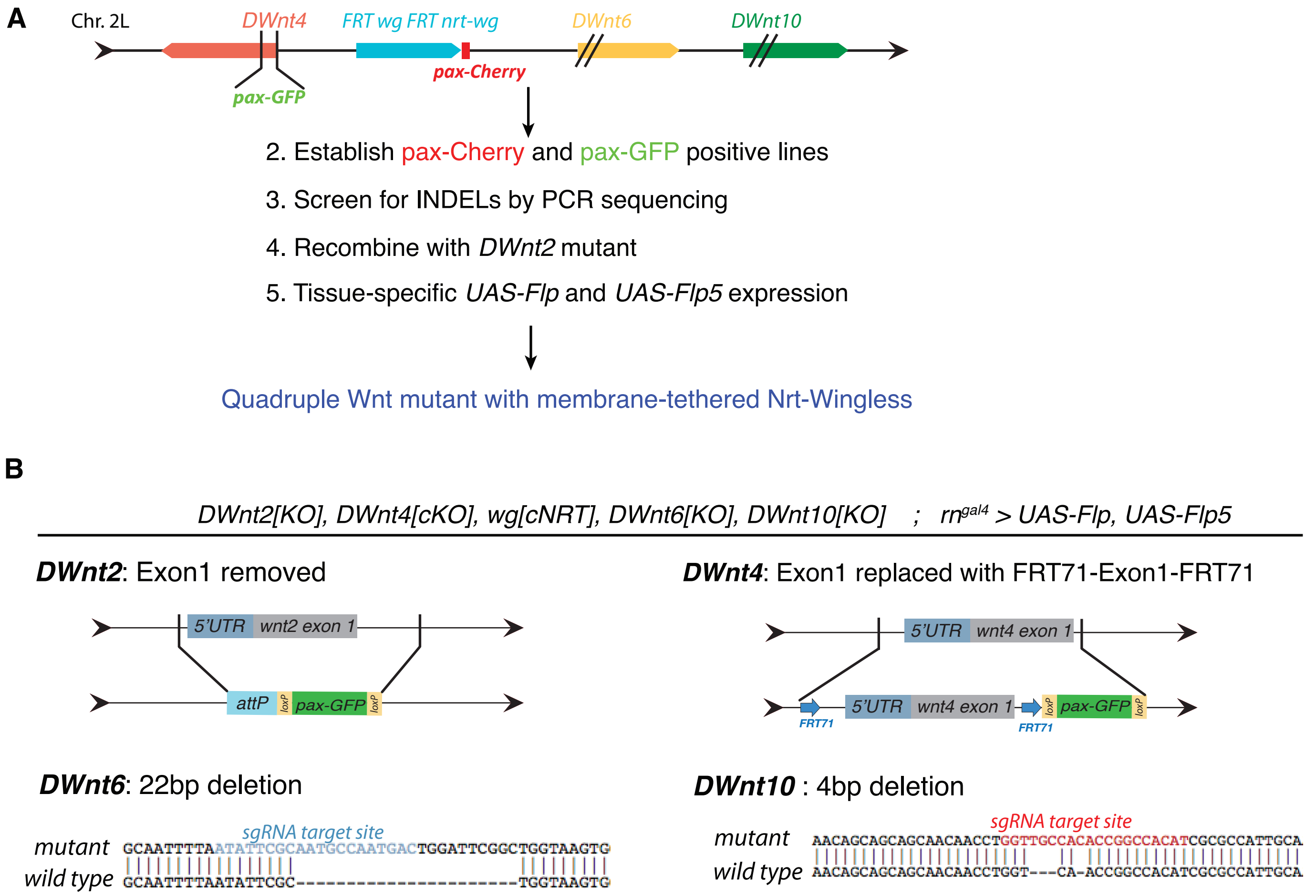

Supplementary Figure 3. Growth and PCP activities of Dsh can be temporally separated (Related to main Figure 3)

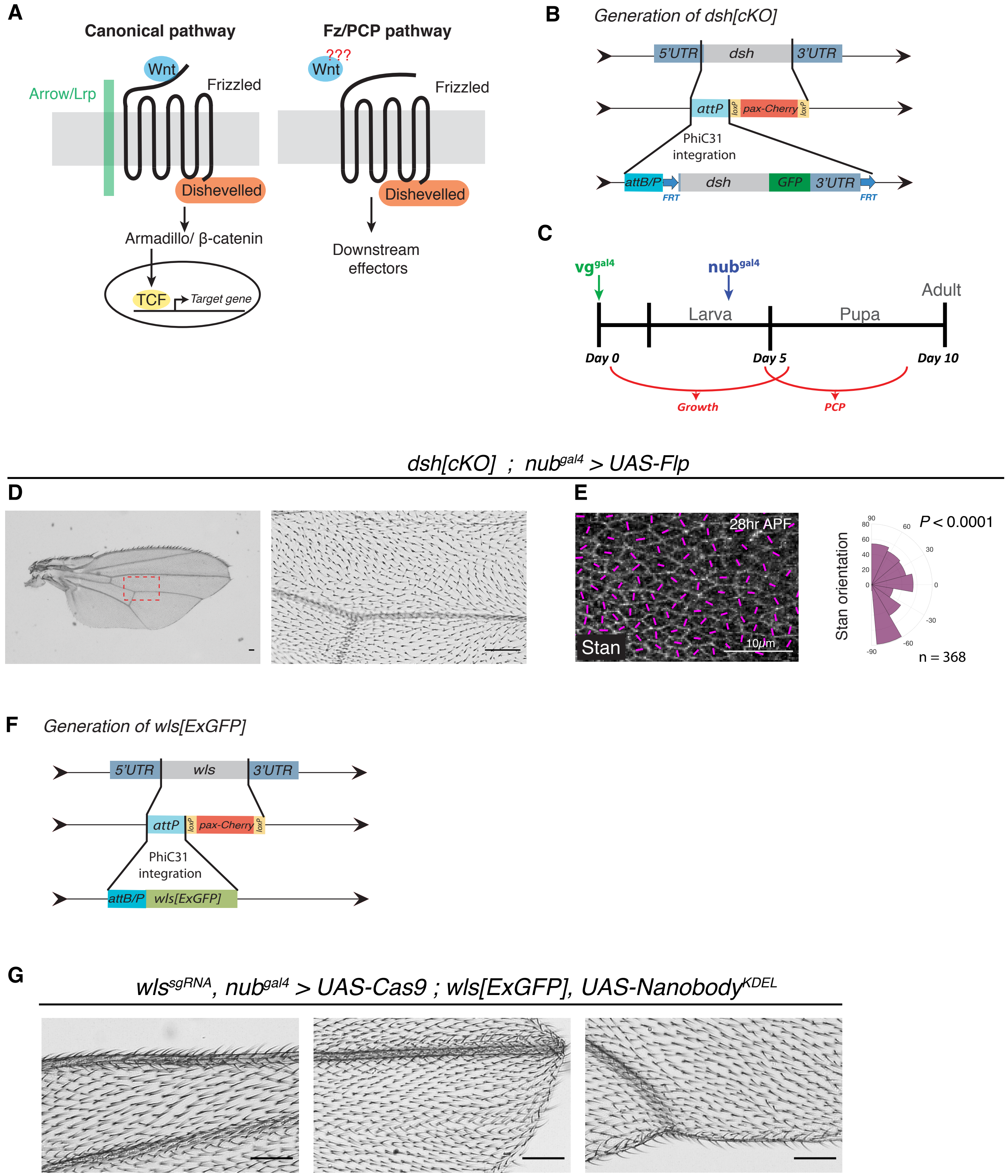

***Supp Fig. 1**: Reporters of DWnt expression (related to Figure 1D and 2A-B)*

(**A**) Generation of *DWnt4* mutant by removing the first exon (in the *wg[cNRT]* background). CRISPR target sites (vertical black bars) were chosen in unconserved regions flanking the 5'UTR and first exon. Details for cloning strategies of all transgenic lines generated in this paper are described in the Methods section. (**B**) Generation of nlsGFP-expressing reporters for DWnt2, DWnt4, DWnt5, and DWnt6. (**C**) Generation of *DWnt10[HA]*. (**D**) Generation of Gal4 lines for *DWnt10* and *DWntD*. (**E, F**) Expression patterns of *DWnt10[GAL4]* and *DWntD[GAL4]*, as reporter with *UAS-GFP* expression. Scale bars are 50μm.

***Supp Fig 2:** A strain for the manipulation of five DWnt genes simultaneously (related to main Figure 2C-D)*

(**A**) Schematic diagram of the generation of a quadruple DWnt mutants in the *wg[cNRT]* background. The *wg[cNRT]* allele was marked with *pax-Cherry*, which allowed us to exclude multi-genic deletions. The conditional DWnt4 mutant was marked with *pax-GFP*, another selection marker. (**B**) Details of the mutations generated in each *DWnt*. INDELS were created in *DWnt6* and *DWnt10*; the first exon of *DWnt2* was removed; the first exon of DWnt4 was flanked by FRT71 sites, which allowed the tissue specific Flp-excision of the region to create a *DWnt4* mutant.

Supp Fig. 3: *Growth and PCP activities of Dsh can be temporally separated (related to main Figure 3)*

(**A**) Comparison of the canonical Wnt pathway and the Fz/PCP pathway. Fz and Dsh are common components of both pathways. (**B**) Schematic of the generation of the Dsh conditional null allele (*dsh[cKO]*). (**C**) A timeline of Drosophila development showing the approximate periods when growth occurs, and PCP is specified. The time of activation of relevant wing drivers is also shown. (**D, E**) Adult wing and pupal wing Stan polarization upon inactivation of *dsh[cKO] with* nub^{gal4} and *UAS-Flp*. (**F**) Generation of *wls[ExGFP]*, with GFP inserted into the fourth extracellular loop of Wls. (G) Various regions of the same wing showing occasional hair mis-orientation adjacent the edge of the wing.

Supplementary Table 1 (Related to Methods Details)

**Oligonucleotides**	SOURCE	IDENTIFIER
DWnt4[KO] 5'arm forward: CATTAGAATTCAGCGCGTCTAAATGGACAC	This paper	N/A
DWnt4[KO] 5'arm reverse: TAATGGGTACCCTCAAATGCTATTATAATTCTGAAAAGTTTATAATAATC	This paper	N/A
DWnt4[KO] 3'arm forward: CATTAACTAGTGCAATCTATAGGTGACTTTAACAATCAG	This paper	N/A
DWnt4[KO] 3'arm reverse: TAATGACGCGTTGTGTATGTTTTCTCCTCCG	This paper	N/A
DWnt2 nlsGFP reporter 5'arm forward: CCCGGGCTAATTATGGGGTGTCGCCCTTCGCTCAGTTCTTATACGACACTCGCACC	This paper	N/A
DWnt2 nlsGFP reporter 5'arm reverse: GCTCACTACTTTCCGCTTCTTCTTAGGCATCGAGCCCAGCAGCTGGCACTAT	This paper	N/A
DWnt2 nlsGFP reporter 3'arm forward: TGCGGCGACGTGGAGGAGAACCCCGGCCCCATGTGGAAAATACATAACAAGCTCTTAATC	This paper	N/A
DWnt2 nlsGFP reporter 3'arm reverse: GCCCTTGAACTCGATTGACGCTCTTCGGGGAGTTACAAAACTCGTTTAATAATATGTATC	This paper	N/A
DWnt4 nlsGFP reporter 5'arm forward: CCCGGGCTAATTATGGGGTGTCGCCCTTCGTGGGAAAGCGTGAAAACTTCTGGCATC	This paper	N/A
DWnt4 nlsGFP reporter 5'arm reverse: GCTCACTACTTTCCGCTTCTTCTTAGGCATGAAGCGGTTGTTGGTGAATTGTGGATT	This paper	N/A
DWnt4 nlsGFP reporter 3'arm forward: TGCGGCGACGTGGAGGAGAACCCCGGCCCCATGCCATCGCCAACGGGAGTCTTT	This paper	N/A
DWnt4 nlsGFP reporter 3'arm reverse: GCCCTTGAACTCGATTGACGCTCTTCGGGGGATCTGACTTGTTCTAGCCATAAATTGTCG	This paper	N/A
DWnt5 nlsGFP reporter 5'arm forward: CCCGGGCTAATTATGGGGTGTCGCCCTTCGGGTATCTATTATTCGAGGTGGATTAGTG	This paper	N/A
DWnt5 nlsGFP reporter 5'arm reverse: AACAATACTCTGTAATAGTAATAGTAAGAGATGCCTAAGAAGAAGCGGAAAGTAGTGAGC	This paper	N/A
DWnt5 nlsGFP reporter 3'arm forward: TGCGGCGACGTGGAGGAGAACCCCGGCCCCATGAGTTGCTACAGAAAAAGGCACTTTCT	This paper	N/A
DWnt5 nlsGFP reporter 3'arm reverse: GCCCTTGAACTCGATTGACGCTCTTCGGGGGTGTCTCGTTGAGCAGTATCACCTG	This paper	N/A
DWnt6 nlsGFP reporter 5'arm forward: CCCGGGCTAATTATGGGGTGTCGCCCTTCGCAGATCCATATGAACGGTCAGCGC	This paper	N/A
DWnt6 nlsGFP reporter 5'arm reverse:	This paper	N/A

GCTCACTACTTTCCGCTTCTTCTTAGGCATAGCCGCCAAATGCAGCGGCAACAA		
DWnt6 nlsGFP reporter 3’arm forward: TGCGGCGACGTGGAGGAGAACCCCGGCCCCATGCGTTTGCTCATGGTAATTGCAATTTTA	This paper	N/A
DWnt6 nlsGFP reporter 3’arm reverse: GCCCTTGAACTCGATTGACGCTCTTCGGGGCCTCCGCGGTTTGTTTATTTATTTATTCAT	This paper	N/A
DWnt10[KO]^{attP} 5’arm forward: CATTAGAATTCAGCTCGGCTCTCGTCTCATAG	This paper	N/A
DWnt10[KO]^{attP} 5’arm reverse: TAATGGGTACCAAAGCATGGAAATTAAGGGAAATATAC	This paper	N/A
DWnt10[KO]^{attP} 3’arm forward: CATTAGACGTCAGAAGATGTTCAGAGGCAGAC	This paper	N/A
DWnt10[KO]^{attP} 3’arm reverse: TAATGACCGGTTAACTAAAACTAAGGCGATGTACTCATG	This paper	N/A
DWnt10[HA] forward: CATTAGAATTCTCATAGTCGCGCGACTCG	This paper	N/A
DWnt10[HA] reverse: TAATGGGTACCGACAGACTAATTATGCATCTTATTGTTCGTATTTACAAAATATATTT	This paper	N/A
DWntD[KO]^{attP} 5’arm forward: CATTAGCGGCCGCATGTAGTGGGGGAATCTCAAG	This paper	N/A
DWntD[KO]^{attP} 5’arm reverse: TAATGGGTACCACACTTATATAGCCTGCAAATCCC	This paper	N/A
DWntD[KO]^{attP} 3’arm forward: CATTAGACGTCCTACAGGTGGTTTAATAATTACGGATTTTAGAGT	This paper	N/A
DWntD[KO]^{attP} 3’arm reverse: TAATGACGCGTCTGATGCAGCGGCCAC	This paper	N/A
DWnt4[cKO] 5’arm forward: GCGAATTGGAGCTGAATTCCTGTAGATAAACCCTTGGGG	This paper	N/A
DWnt4[cKO] 5’arm reverse: TCTAGAAAGTATAGAAACTTCAGGTGACTTTAACAATCAGGTGATTG	This paper	N/A
DWnt4[cKO] exon1 forward: GAAGTTTCTATACTTTCTAGAGAATAGAAACTTCATCGATTGCATTTTGCTCCTAATATCTTCAATTTCCACACATTTTTCATTTGAGA	This paper	N/A
DWnt4[cKO] exon1 reverse: GTTGGGGCACTACGGGTACCGAAGTTTCTATTCTCTAGAAAGTATAGAAACTTCAGCATTTGAGGACGGCTAACATGAAATCAATATTTTCGATTTCTAGAATTTTGCC	This paper	N/A
DWnt4[cKO] 3’arm forward: CATTAACTAGTATTATAATTCTGAAAAGTTTATAATAATCGCTTCCTG	This paper	N/A
DWnt4[cKO] 3’arm reverse: TAATGACGCGTGCTTCAAGCTTATACGCACAGAA	This paper	N/A
DWnt2[KO] 5’arm forward: CATTAGCGGCCGCATTATCTGGAATTTAAAAGTCTTGGCACAC	This paper	N/A
DWnt2[KO] 5’arm reverse: TAATGGGTACCGTACTACTACTATCCAACTTTCCACTCT	This paper	N/A

DWnt2[KO] 3’arm forward: CATTAACGCGTGTATTTTGATTTTGGTGAAATGCTTGAAAAG	This paper	N/A
DWnt2[KO] 3’arm reverse: TAATGACCGGTCATAAAGGCTGTTCAACATGCC	This paper	N/A
dsh[KO]^{attP} 5’arm forward: ATGCAGGCGGCCGCTATCAGCCGTCGTGCGTG	This paper	N/A
dsh[KO]^{attP} 5’arm reverse: TGACTCACATATGCCTCGACGCGATAAAACGCGATC	This paper	N/A
dsh[KO]^{attP} 3’arm forward: TGCACCTAGGTGAGATTGGTTTCTTTAACGCATTTTAGCTG	This paper	N/A
dsh[KO]^{attP} 3’arm reverse: CGTCAACCGGTCATTTCGTATTAGTTGTATGTACGGAAGTTGAC	This paper	N/A
RIV10$^{dsh\text{-}GFP}$ forward: ATGGTCAGCTAGCGATCGCGTTTTATCGCGTCGAGGAGTTTTCCCGTGGATTTCCGCAGTCGGCGCAGCTAAA	This paper	N/A
RIV10$^{dsh\text{-}GFP}$ reverse: TCCATGACCGGTCCATGCCCGGAATGTTTCCG	This paper	N/A
pBS-KS$^{dsh\text{-}GFP}$ forward: CGTATCCGTATTTAATTACGTATTGGGAGGTTCGGGAGGTGGTTCGGGAGGTGGAGGTTCGGGAGTGAGCAAGGGCGAGGA	This paper	N/A
pBS-KS$^{dsh\text{-}GFP}$ reverse: GAAAGGTTTCTACAATACgtaCTTGTACAGCTCGTCCATGCC	This paper	N/A
wls[ExGFP] forward: TGACGACGTCGTGAGCAAGGGCGAGGAGCTGTTCA	This paper	N/A
wls[ExGFP] reverse: GTCGACGTCCTTGTACAGCTCGTCCATGCCGAG	This paper	N/A
